# Hybrids of Salicylalkylamides and Mannich Bases: Control of the Amide Conformation by Hydrogen Bonding in Solution and in the Solid State

**DOI:** 10.3390/molecules20011686

**Published:** 2015-01-20

**Authors:** Christian Dank, Barbara Kirchknopf, Matthias Mastalir, Hanspeter Kählig, Susanne Felsinger, Alexander Roller, Vladimir B. Arion, Hubert Gstach

**Affiliations:** 1 Institute of Medical Chemistry, Center of Pathobiochemistry and Genetics, Medical University of Vienna, Währingerstrasse 10, Vienna 1090, Austria; E-Mails: christian.dank@univie.ac.at (C.D.); matthias.mastalir@gmx.at (M.M.); 2 Institute of Organic Chemistry, University of Vienna, Währingerstrasse 38, Vienna 1090, Austria; E-Mails: hanspeter.kaehlig@univie.ac.at (H.K.); susanne.felsinger@univie.ac.at (S.F.); 3 University of Applied Sciences Wiener Neustadt, Konrad-Lorenz-Strasse 10, Tulln a. d. Donau 3430, Austria; E-Mail: barbara.kirchknopf@gmx.at; 4 X-ray Structure Analysis Centre, University of Vienna, Währingerstrasse 38, Vienna 1090, Austria; E-Mails: alexander.roller@univie.ac.at (A.R.); vladimir.arion@univie.ac.at (V.B.A.)

**Keywords:** salicylamide, Mannich base, hybrid, conformation, intramolecular hydrogen bonding, conformational switch

## Abstract

3-Aminomethylation of salicylalkylamides afforded hybrids with a Mannich base. In addition, it triggered the rotation of the amide bond. The observed conformational switch is driven by strong intramolecular hydrogen bonding between the Mannich base and phenolic group. Crystal structure analysis reveals the stabilization of the hybrid molecules by double hydrogen bonding of the phenolic OH, which acts as an acceptor and donor simultaneously. The molecules contain an amide site and a Mannich base site in an orthogonal spatial arrangement. The intramolecular hydrogen bonds are persistent in a nonpolar solvent (e.g., chloroform). The conformational change can be reversed upon protection or protonation of the Mannich base nitrogen.

## 1. Introduction

Salicylic acid amides are privileged scaffolds in medicinal chemistry. Pharmacological activities have been reported for numerous *N-*aryl (anilides) and *N-*alkyl salicylamides in the fields of antimicrobials [1,2,3,4,5,6,7,8,9,10,11,12], antivirals [13,14,15,16,17], anthelmintics [18,19], antimalarials [20,21,22,23], antimycotics [24], molluscicides [25,26], as well as target-specific interactions [27,28,29,30,31,32,33]. The conformations of salicylamides in solution and in the solid state are controlled by inter- and intramolecular networks of different types of hydrogen bonding. The biological activities of salicylamides, as well as their specificity are connected to these conformational features [34,35,36]. Control over the conformations of the salicylamide scaffold is of great value in medicinal chemistry. In this contribution, we report triggering the conformation of salicylalkylamides by intramolecular competition for hydrogen bonding.

**Figure 1:**
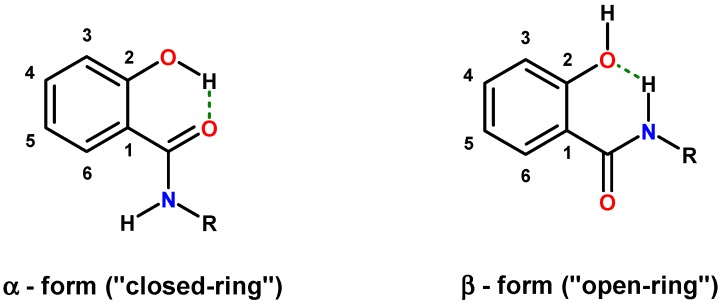
Intramolecular hydrogen bonding modes in salicylamides.

Intramolecular hydrogen bonding in salicylamides creates two distinct conformations: the α-form (“closed-ring”) with the phenol as the *H*-donor and the amide oxygen as the *H*-acceptor (O*H*···*O*=CNH); and the β-form (“open-ring”) with the amide-N*H* as a donor and the phenolic oxygen as an acceptor (CON*H*···*O*H) (Figure 1).

Both hydrogen bonding modes (α-form: O*H*···*O*=CNH; β-form: CON*H*···*O*H) (Figure 1) annulate six-membered rings onto the scaffold. Both structures are fairly planar and rigid. Such motifs stabilized by intramolecular hydrogen bonding mimic flat aromatic or heteroaromatic systems found in many drugs [37,38,39,40,41,42]. Salicylamide scaffolds with stabilized conformation have been applied in the design of helix mimetics and the control of conformation in foldamers, respectively [43,44].

**Figure 2:**
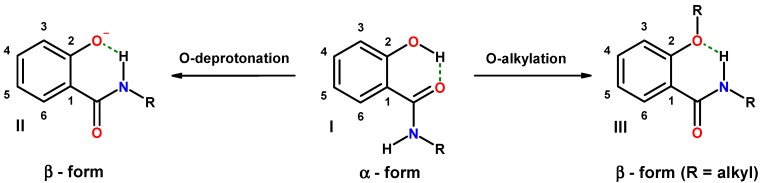
Chemically induced conformational switches of secondary salicylamides from the α- to the β-form (dashed lines: hydrogen bonding).

*N*-monosubstituted 2-hydroxybenzamides prefer the α-form in solution, which can be switched to the β-form by chemical manipulation. Deprotonation of the phenolic group [45,46,47,48] or substitution through alkylation [49] removes the hydrogen atom necessary for the stabilization of the α-form. Concomitantly, the hydrogen acceptor strength of the oxygen atom (oxyanion or alkoxy group) is enhanced. Upon rotation of the amide function, the alternative hydrogen bonding between the phenolate oxygen and amide hydrogen can be established. The result is a switch from the α-form (**I**) to the β-form ((**II**) and (**III**) in Figure 2).

**Figure 3:**
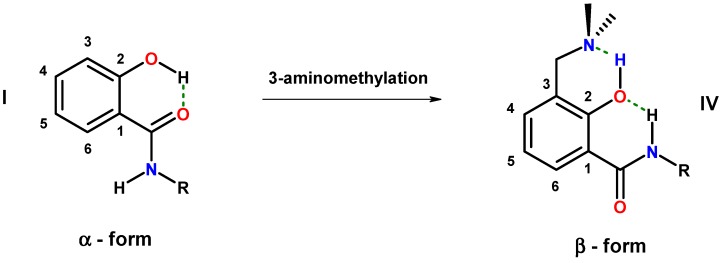
Competition for intramolecular hydrogen bonding in hybrids of salicylalkylamides and Mannich bases (dashed lines: hydrogen bonding).

Another possibility to control the conformation is 3-aminoalkylation of salicylamides, which introduces a basic functionality with an available nitrogen lone pair able to compete with the amide oxygen for the acidic hydrogen of the phenolic group. Such a reaction affords a hybrid composed of salicylamide and a Mannich base. The presence of a basic nitrogen of the 3-aminomethyl residue is in ideal premise for the formation of an intramolecular hydrogen bond to the phenolic hydrogen, which should trigger the conformational change from the α- to the β-form (Figure 3, **IV**).

Intramolecular hydrogen bonding in Mannich bases has been extensively investigated by different techniques [50,51,52,53,54] and, in particular, by NMR spectroscopy [55,56]. Furthermore, introduction of a Mannich base into position 3 of salicylamides creates an aminoalcohol motif, which is a recognized pharmacophore in drugs (e.g., antimalarials).

## 2. Results and Discussion

### 2.1. Hybrids of Salicylalkylamides and Mannich Bases

**Scheme 1:**
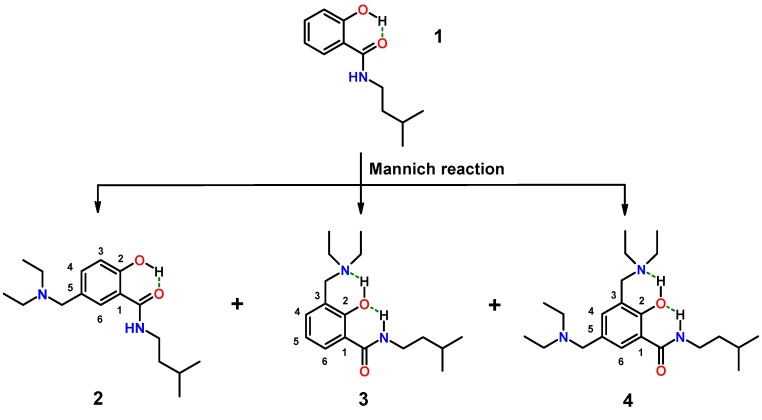
Mixtures of salicylalkylamide-Mannich base hybrids (**2**–**4**) upon aminomethylation of salicylamide **1**.

Synthesis of hybrids composed of salicylamide and a Mannich base started with aminomethylation of 2-hydroxy-*N-*(3-methyl-butyl)-benzamide (**1**) (Scheme 1; the isopentyl residue was chosen to mimic an isoprene moiety). The applied Mannich reaction is a widely-used standard C-C bond-forming procedure in organic chemistry [57]. The electrophilic substitution of salicylamides with iminium ions affords *N*-, as well as *C*-Mannich bases, depending on the substrate used and the reaction conditions applied. Parent salicylamide yields *N-*Mannich bases, which have been investigated as models for prodrug systems to deliver in water sparingly soluble drugs into systemic circulation [58,59]. Salicylamides bearing more than one hydroxy group, such as β-resorcylic acid amide or gentisic acid amide, afford *C*-Mannich bases upon aminomethylation [60], as well as niclosamide [61]. From the reaction of **1** with diethyl amine and formaldehyde, no *N-*Mannich base was obtained, although the reaction starts on the amide nitrogen [62]. This is in line with our observation that the corresponding ester congener of **1** (isopentyl ester) did not give any product under the conditions applied for the syntheses of **2**–**4**.

Formation of mixtures of *C*-Mannich bases is programmed due to unsubstituted 3- and 5-positions in **1**. Both positions are amenable to electrophilic attack by iminium ions. We attempted to isolate all possible isomers by offering one equivalent of amine for the reaction. Only isomers **3** and **4** contain *N*-diethylaminomethyl residues in position 3 of the salicylamide scaffold, which would be mandatory for intramolecular hydrogen bonding of the acidic phenol-hydrogen to the basic nitrogen of the Mannich base. The nitrogen of the 5-aminomethyl residue in isomer **2** is expected to form intermolecular hydrogen bridges. The synthesis is depicted in Scheme 1.

Aminomethylation of **1** with diethyl amine (free base) and formaldehyde worked without the addition of acid in a protic solvent (ethanol). For the separation of the basic products **2**–**4**, sequential preparative chromatographic separations had to be applied. Finally, all three isomers were obtained from one experiment in sufficient purity for analytical characterization. The structures of **2**–**4** were verified by 1D and 2D NMR spectroscopy (atom numbering for resonance assignment is shown in Scheme 1).

Isomer **2** is characterized by only one coupling of *H*-6 over four bonds to *H*-4 (δ 7.29 ppm, *^4^J* = 1.6 Hz). *H*-3 of **2** is recorded as a doublet at δ 6.89 ppm with a typical *ortho*-coupling constant of *^3^J* = 8.5 Hz. Isomer **3** revealed the aromatic *H*-6 at δ 8.08 ppm as a doublet of doublets with coupling constants of *^3^J* = 7.9 Hz and *^4^J* = 1.5 Hz, which is indicative of unsubstituted 4- and 5-positions. In addition, the exocyclic methylene protons of the aminomethyl substituent showed a strong cross-peak to *C*-2 of the phenyl ring in the HMBC spectrum, which is in agreement with the substitution in position 3. The structure of the 3,5-bis-diethyl aminomethyl derivative **4** is proven by a doublet at δ 7.07 ppm (*^4^J* = 2.8 Hz) for *H*-4 and a second doublet at δ 7.90 ppm (*^4^J* = 2.8 Hz) for *H*-6 (the full assignment of NMR resonances is provided in the Supporting Information).

#### 2.1.1. Assessment of the Conformation of Salicylalkylamides in Chloroform-d_1_ by NMR Spectroscopy

**Figure 4:**
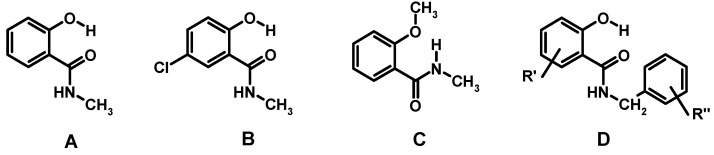
Structures of compounds **A**, **B**, **C**, and **D**.

The conformation of salicylalkylamides in solution can be determined by NMR spectroscopy. The distinctive chemical shift changes (δ) revealed by NMR for the transition from the α- into the β-form will be briefly discussed in the following. Our starting material **1** is comparable to 2-hydroxy-*N*-methyl-benzamide (**A**, Figure 4), the parent *N*-alkyl salicylalkylamide. Compound **A**, as well as 5-chloro congener **B** have been shown to adopt the α-conformation in 1,2-dichloroethane, as well as in DMSO by emission spectroscopy. Large Stokes shifts were indicative of excited-state intramolecular proton transfer, which is in good agreement with strong intramolecular hydrogen bonding [63]. We inspected the ^13^C/^1^H-NMR (CDCl_3_) spectra reported for **A**, **B** [63,64], as well as for an additional 25 *N*-benzyl salicylamides **D** [34]. We found out that the α-form of salicylalkylamides reveal δ < 7 ppm for amide-N*H*, δ 6.8–7.5 ppm for aromatic *H*-6 (outliers from [34] removed, e.g., 5-NO_2_) and δ 11.5–12.6 ppm for phenolic 2-O*H*, which appears mainly as a sharp singlet. It has to be noted that investigation of hydrogen bonding by NMR is very sensitive to the polarity of the solvent used, the sample concentration, the water content and temperature, respectively. Further spectroscopic and structural data with regard to 2-hydroxybenzamides are available [65].

2-Methoxy-*N-*methylbenzamide (**C**) exemplifies the removal of the α-form stabilizing phenolic hydrogen from parent *N*-alkyl salicylamide **A** upon alkylation [63,66]. Compound **C** has been shown to contain an intramolecular H-bond by fluorescence spectroscopy [49], which can only be ascribed to the presence of the β-form in solution. Derivatives of **C** have received much attention for design of arylamide oligomers [67,68] and supramolecular recognition units [69]. *Ab initio* methods revealed a persistent intramolecular hydrogen bond between the 2-methoxy group and N*H* of the amide in a nonpolar environment, such as chloroform. In protic solvents (methanol, water), the intramolecular H-bond is disturbed by interaction with the solvent [70]. Significant changes of chemical shifts in the NMR spectra (CDCl_3_) reflect the conformational switch. In the β-form of **C**, the amide-N*H*, as well as *H*-6 reveal downfield shifts to δ 7.8 and δ 8.2 ppm, respectively (Table 1). The observed downfield shift for amide-N*H* in the β-form is in agreement with intramolecular hydrogen bonding. Deshielding of *H*-6 is probably due to the closer proximity of *H*-6 to the amide carbonyl in the β-form. Both conformers α and β are fairly planar with respect to amide and the aromatic ring and are quite rigid due to intramolecular hydrogen bonding [70]. The amide carbonyl is *exo* to *H*-6 in the α-form, whereas it is *endo* in the β-form. Therefore, *H*-6 is spatially closer to the amide carbonyl in the β-conformation. The magnetic anisotropy of the amide carbonyl contributes to downfield shift of *H*-6 in the β-form, similar to the effects found for carbonyl compounds [71,72,73,74,75,76,77]. Alternatively, electric field effects, as well as orbital interactions have to be taken into account for the explanation of the observed shift change [78].

**Table 1:** Selected ^13^C/^1^H chemical shifts (δ) of literature compounds **A**, **B**, **C**, and **D**.

^13^C{^1^H} NMR (CDCl_3_)								^1^H-NMR (CDCl_3_)			
ID	C-1	C-2	C-3	C-4	C-5	C-6	C=O	H-6	NH	2-OH	Ref.
**A**	114.6	161.8	118.8	134.5	119.0	125.7	171.1	7.4	6.8	12.4	[63,64]
**B**	115.3	160.1	120.2	134.0	123.3	124.9	169.5	7.3	6.2 ^b^	12.1 ^s^	[63]
**C**	121.1	157.3	111.1	132.5	121.4	132.1	165.9	8.2	7.8	^_^	[63,66]
**D**	*	*	*	*	*	*	*	6.8–7.5	<7	11.5–12.6	[34]

(* not assigned; s, sharp; b, broad).

Furthermore, also ^13^C chemical shifts are indicative of the conformational switch from the α- to the β-form. The 2-hydroxy congeners **A** and **B** adopt the α-conformation in a nonpolar environment. The phenolic hydrogen of **A** and **B** is in the ideal position for the formation of intramolecular resonance-assisted hydrogen bonding (RAHB) to the oxygen of the amide group [79]. ^13^C chemical shifts are sensitive to mesomeric effects. The RAHB established in the α-form can be described by quasi-aromatic resonance structures resembling *C*-1 and *C*-2 quinoid forms (see Supporting Information, Figure S1) with less delocalization of the nitrogen lone pair into the amide carbonyl [80]. Consequentially, in the α-form, the ^13^C resonance for *C*-1 of **A** and **B** should experience an upfield shift and the ^13^C resonance for *C*-2 and the carbonyl a downfield shift compared to the β-form. Analysis of the shift changes for *C*-1, *C*-2, and *C*=O of **A** and **B** and those found for 2-methoxy derivative **C** shows the expected shift changes as discussed above (Table 1). Compound **C** reveals for the carbon resonance of the carbonyl group and for *C*-2 an upfield shift compared to **A** and **B**, whereas *C*-1 experiences a downfield shift, respectively. In addition, both carbons *C*-6 and *C*-4 are in *meta*-position to the 2-substituents, OH for **A**, **B** and OCH_3_ for **C**. *Meta*-positions are the least affected by the mesomeric effects of the substituents. Nevertheless, *C*-6 reveals a downfield shift by ~6.8 ppm for the β-form of **C**, whereas *C*-4, in contrast, an upfield shift by ~2 ppm. This opposite shift change displayed for *C*-4 and *C*-6 is also connected to the conformational switch.

#### 2.1.2. Assessment of the Conformation of Hybrids **2**–**4** in Chloroform-d_1_ by NMR Spectroscopy

For the discussion of the conformation adopted by isomers **2**–**4**, selected ^13^C/^1^H chemical shifts (δ) of **1**–**4** in chloroform-*d*_1_ are summarized in Table 2.

Comparing the ^1^H/^13^C δ-values of isomers **2**–**4** with the one recorded for the α-form of the starting material **1** (Table 2) showed significant differences. Compound **2**, the 5-aminomethyl-substituted isomer revealed very similar δ for amide-N*H*, as well as for *H*-6 of the aromatic ring compared to **1**, suggesting also an “α-form” for **2**. The phenolic hydrogen of **2** was not detectable in the spectrum, due to a rapid exchange (most probably an intermolecular exchange driven by the basic aminomethyl moiety in position 5). However, isomers **3** and **4**, both bearing a diethylaminomethyl residue in position 3, have changed the conformation in chloroform-*d*_1_ with respect to salicylamide **1**. Most remarkable is a large downfield shift of the amide proton resonance by approximately 2 ppm, indicating a strong intramolecular N*H*···*O*H hydrogen bonding interaction. In addition, the *H*-6 resonance is downfield shifted due to a deshielding effect of the amide group. The ^1^H-NMR spectra reveal that both compounds **3** and **4** adopt a β-form conformation in chloroform-*d*_1_. This is consistent also with the shift changes of the ^13^C resonances of **3** and **4** with respect to those of **1**: (*C*=O) Δ(δ) ~−4, (*C*-6) Δ(δ) ~+5 and (*C*-1) Δ(δ) ~+5 ppm.

**Table 2:** Selected ^13^C/^1^H chemical shifts (δ) of compounds **1**–**4**.

^13^C{^1^H} NMR (400 MHz, CDCl_3_)								^1^H-NMR (400 MHz, CDCl_3_)		
**ID**	**C-1**	**C-2**	**C-3**	**C-4**	**C-5**	**C-6**	**C=O**	**H-6**	**NH**	**2-OH**
**1**	114.5	161.6	118.6	134.2	118.7	125.4	170.0	7.4	6.4	12.4 ^s^
**2**	114.4	160.5	118.2	135.0	129.6	125.8	170.0	7.4	6.5	nd
**3**	119.9	158.2	122.0	131.3	118.7	130.8	166.1	8.1	8.6	7.5–9.5
**4**	119.0	156.9	122.1	132.0	129.5	131.0	166.1	7.9	8.5	10.5–11.5

(nd, not detectable; s, sharp).

Our findings suggest that the intramolecular hydrogen bonding within the Mannich base motif in hybrids **3** and **4** is indeed established and favored over the alternative O*H*···*O*=CNH bonding present in starting material **1** and 5-aminomethylated congener **2**. In addition, the formation of a phenolate anion via deprotonation of the phenolic group by the Mannich base nitrogen can be excluded taking into account an upfield shift of *C*-2 (Δ(δ) ~2–3 ppm found for hybrids **3** and **4**. Phenolate carbon 2 would reveal a tremendous downfield shift of at least 10 ppm. The conformation in the solid state will be discussed in Section 2.2.

#### 2.1.3. Synthesis of a Small Library of Hybrids of Mannich Bases and Salicylalkylamide **5**

**Scheme 2:**
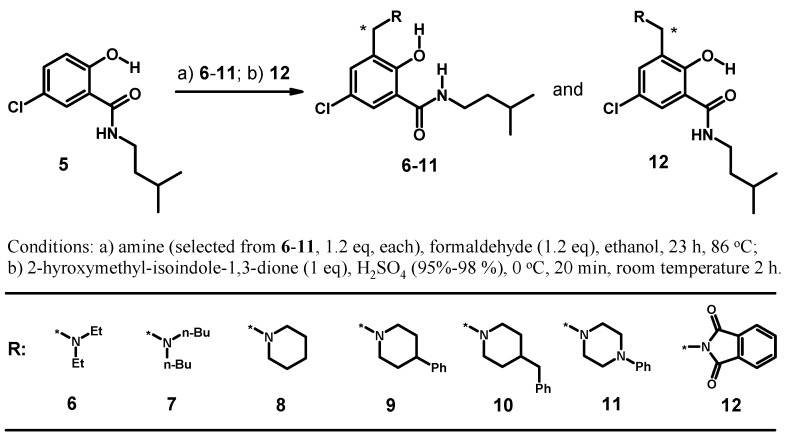
Synthesis of a small library of salicylamide-Mannich base hybrids.

We extended the investigations by the synthesis of a small library of hybrids using different secondary amines for the Mannich reaction with **5**. The synthesis is depicted in Scheme 2.

The 5-chloro substitution was chosen for several reasons. Blocking position 5 might facilitate the work-up by the suppression of isomers and concomitantly increasing the yield of the desired hybrids, **6**–**12**. Additionally, more drug-like molecules are obtained by metabolic stabilization of the scaffold. Full characterization of compounds **6**–**12** by NMR spectroscopy and analytical data is provided in the Supporting Information.

#### 2.1.4. Assessment of the Conformation of Hybrids **6**–**12** in Chloroform-d_1_ by NMR Spectroscopy

**Figure 5:**
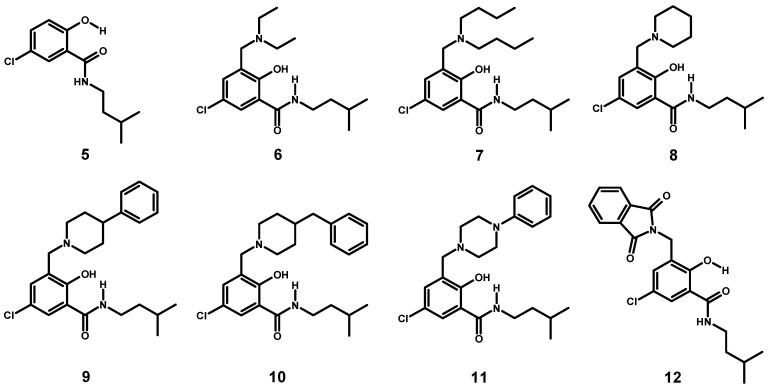
Structures of compounds **5**–**12**.

Selected chemical shifts useful for the assessment of the conformation adopted by **6**–**12** in comparison to **5** are summarized in Table 3 (for a discussion of **12**, see Section 2.1.5). The molecular structures are depicted in Figure 5.

**Table 3:** Selected ^13^C/^1^H chemical shifts (δ) of compounds **5**–**12**.

^13^C{^1^H} NMR (400 MHz, CDCl_3_)								^1^H-NMR (400 MHz, CDCl_3_)		
**ID**	**C-1**	**C-2**	**C-3**	**C-4**	**C-5**	**C-6**	**C=O**	**H-6**	**NH**	**2-OH**
**5**	115.5	160.2	120.3	134.1	123.4	125.0	169.0	7.2	6.2	12.3 ^sharp^
**6**	121.3	157.1	123.6	130.8	123.4	130.2	164.8	8.0	8.6	10.8–11.8
**7**	121.3	157.0	123.7	130.8	123.4	130.2	164.8	8.0	8.6	10.5–11.5
**8**	121.2	156.6	123.2	131.0	123.7	130.3	164.8	8.0	8.5	9.7–10.7
**9**	121.3	156.2	123.4	131.1	123.9	130.3	164.7	8.1	8.4	11.0–12.0
**10**	121.2	156.4	123.3	130.9	123.7	130.2	164.7	8.1	8.5	11.2–12.2
**11**	121.1	155.6	123.3	131.4	124.3	130.2	164.6	8.1	8.3	10.8–11.8
**12**	115.5	157.8	126.8	132.4	123.0	124.8	168.7	7.2	6.6	12.7 ^sharp^

Starting material **5** adopts the α-form, as revealed by the very similar ^13^C/^1^H δ-values with respect to **1** and reference compounds **A** and **B**. From the chemical shifts summarized in Table 2, it can be deduced that all novel hybrids **6**–**11** prefer the β-conformation in chloroform-*d*_1_. In particular, this is indicated by downfield shifts of *H*-6 δ-values >8 ppm, a shift-range of δ 8.3–8.6 ppm for CON*H* and broadening of 2-O*H* resonance, respectively. Additional support for the assessment of the β-conformation to hybrids **6**–**11** is obtained from the ^13^C spectra. The recorded changes of δ-values follow the same trend as already discussed for isomers **3** and **4**: (*C*=O) Δ(δ) ~−4, (*C*-6) Δ(δ) ~+5 and (*C*-1) Δ(δ) ~+5 ppm. The conformation adopted in the solid state will be discussed in Section 2.2.

#### 2.1.5. Reversal of the β-Conformation of Salicylalkylamide-Mannich Base Hybrids

The conformational switch of salicylamides (**1**, **5**) from the α- to the β-conformation in hybrids with a Mannich base is triggered by the exocyclic basic nitrogen of the Mannich base. Consequently, blocking the nitrogen in a hybrid structure by a protecting group, which removes the basicity of the nitrogen, should switch back the β-conformation of the hybrid to the α-form of the salicylamide. We demonstrated this by synthesis of *N-*protected derivative **12** (Figure 5). Salicylamide **5** was subjected to a Tscherniac–Einhorn reaction, which installs a non-basic phthalimidomethyl residue in position 3 of the aromatic ring in **5** [81,82]. Such reactions are known with salicylic acids [83]. The NMR spectrum of **12** in chloroform-*d*_1_ revealed δ 7.20 ppm for *H*-6 and δ 6.57 ppm for CON*H*, respectively. These values correspond indeed to the shifts recorded for **5** in the α-conformation.

**Figure 6:**
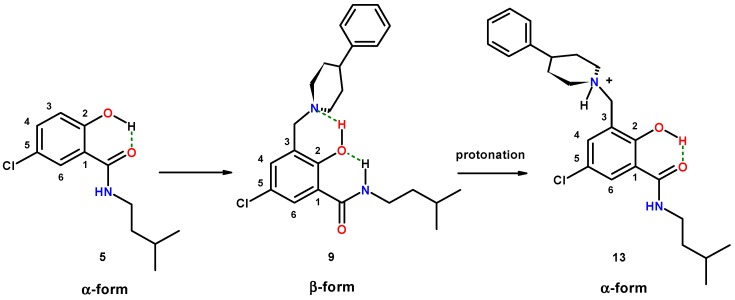
Reversal of the conformational switch in the hybrid of salicylamide-Mannich base **9** upon protonation of the Mannich base nitrogen, affording **13** (dashed lines: hydrogen bonding).

Competition for the basic nitrogen with a strong acid, such as hydrogen chloride, should also destroy the hydrogen bonding of the less acidic phenol and consequently switch back the β-conformation of the hybrids into the α-form of the parent amides. For this purpose, we prepared hydrogen chloride salts of salicylamide-Mannich base hybrids **3**, **6** and **9**, respectively (Figure 6). Unfortunately, NMR inspection in chloroform-*d*_1_ was not feasible, due to the insolubility of the hydrochlorides. We were able to grow X-ray diffraction quality crystals of hydrochlorides of **9** (×HCl: **13**) and **3** (×HCl: **14**). The hydrochloride of 5-chloro substituted derivative **6** (×HCl: **15**) was isolated as a powder of tiny crystals. As expected, at least in the solid state, the conformation switched back from the β- to the α-form for both hydrochlorides **13** and **14** (Figure 6: **13**; for **14**, see Section 2.2.3).

### 2.2. Crystallographic Structure Determination

Crystal structures of parent primary salicylamide (2-hydroxybenzamide) have been reported by Sasada *et al.* [84] and Pertlik [85]. Solid state structures for secondary and tertiary salicylamides are documented in several publications [80,86,87,88,89,90,91]. To the best of our knowledge, there are no crystal structures for hybrids of salicylamide and the Mannich base reported (a substructure search in the database of Cambridge Crystallographic Data Centre (CCDC) did not reveal comparable structures).

#### 2.2.1. *5-Chloro-2-hydroxy-N-(3-methyl-butyl)-3-(4-phenyl-piperidin-1-ylmethyl)-benzamide* (**9**)

**Figure 7:**
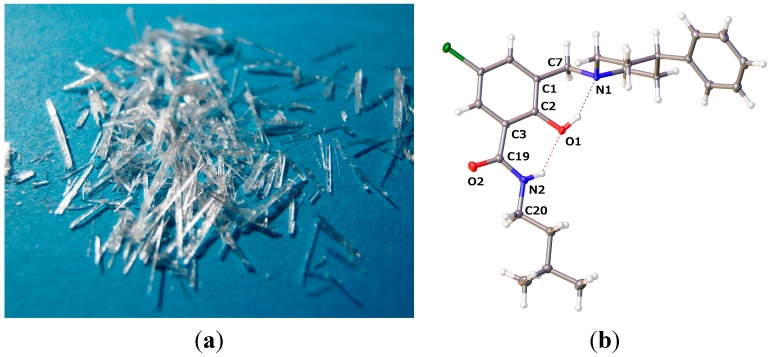
(**a**) Crystals grown for **9**; (**b**) molecular structure of **9** (drawn at a 50% probability level; dashed lines: hydrogen bonding).

The crystals grown for **9** are shown in Figure 7a. The molecular structure is depicted in Figure 7b, while selected geometrical parameters are given in Table 4. The residues of amine and the amide site of **9** are close to orthogonal. Such a scaffold would be able to bind to a target with one site and concomitantly provide a molecular architecture for filling a steep pocket. The nature of the biological information offered in the two diversity sites is a matter of design. Polar, as well as hydrophobic interactions can be built into the orthogonal arms of molecule **9**.

**Figure 8:**
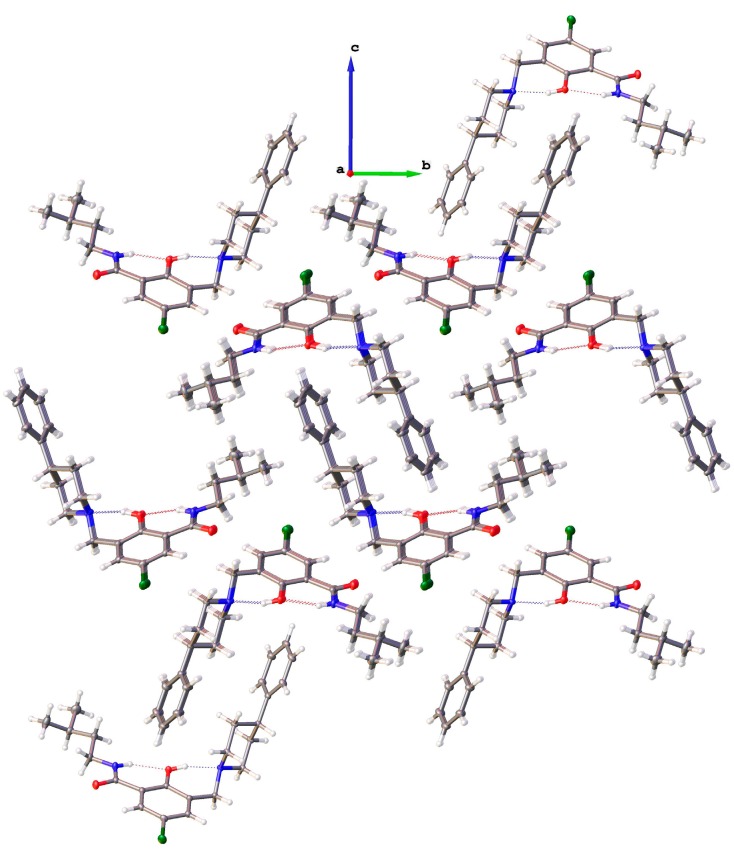
Crystal packing pattern of **9** along the *a*-axis with extended residues showing the hydrophobic contacts and the lack of intermolecular hydrogen bonding.

Molecules of **9** are not involved in intermolecular hydrogen bonding in the crystal, as revealed by the crystal packing pattern (Figure 8). Two intramolecular hydrogen bonds are evident in **9**, namely O1–H1···N1 and N2–H2···O1, with the geometric parameters quoted in Table 5, which force the molecule to adopt the β-conformation. The interactions of the molecules within the crystal are confined to hydrophobic contacts. There are no π-π* interactions between the aromatic fragments of the molecule in the crystal.

**Table 4:** Selected interatomic distances (Å) and torsion angles (°).

	9	13	14
O2–C19	1.2329(12)	1.2546(15)	1.2576(12)
N2–C19	1.3444(13)	1.3289(17)	1.3288(13)
C3–C19	1.5009(13)	1.4952(17)	1.4885(13)
N2–C20	1.4577(13)	1.4630(17)	1.4635(12)
O1–C2	1.3633(11)	1.3469(14)	1.3461(11)
O1–C2–C3–C19	−3.30(15)	4.54(18)	0.49(14)
C2–C1–C7–N1	−48.24(12)	100.91(13)	107.51(10)

**Table 5:** Hydrogen-bond geometry (Å, °) for **9**.

*D–*H···*A*	*D–*H	H···*A*	*D*···*A*	*D–*H···*A*
O1–H1···N1	0.84	1.897	2.6561(11)	149.7
N2–H2···O1	0.88	2.015	2.7120(11)	135.3

#### 2.2.2. *[5-Chloro-2-hydroxy-3-(3-methyl-butylcarbamoyl)-benzyl]-4-phenylpiperidinium Chloride* (**13**)

**Figure 9:**
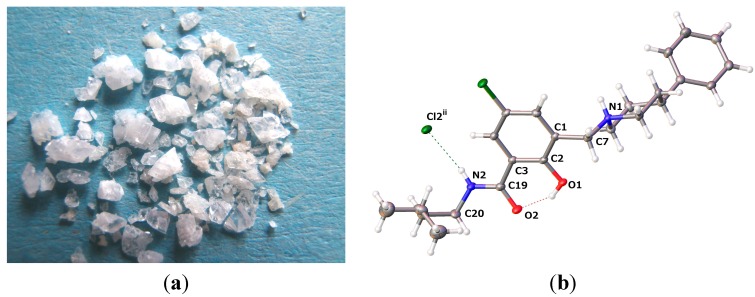
(**a**) Crystals grown for **13**; (**b**) molecular structure of **13** (drawn at a 50% probability level; dashed lines: hydrogen bonding).

The crystals grown for **13** are shown in Figure 9a. The molecular structure of **13** is depicted in Figure 9b, while selected geometrical parameters are quoted in Table 4.

The crystal structure of **13** reveals the change of molecule conformation upon protonation of free base **9**. The lone pair of the nitrogen in the Mannich base is no longer available for the phenolic hydrogen to establish a hydrogen bond. This triggers the β-conformation of hybrid **9** back to the α-form. The two molecules, **9** and **13**, address very different spaces in the pharmacophoric room.

**Figure 10:**
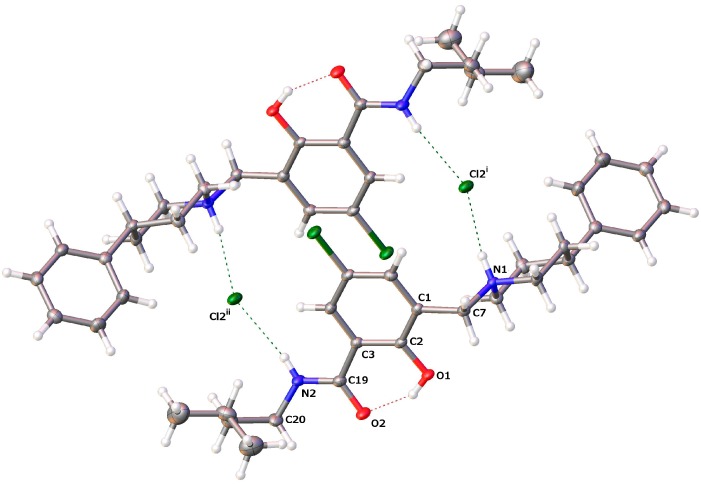
Crystal packing pattern showing intermolecular H-bonding to chloride ions in dimers of **13** (dashed lines: hydrogen bonding).

The crystals of **13** are composed of centrosymmetric dimers in which the monomers are held together by hydrogen bonding and electrostatic interactions. Each protonated molecule in **13** forms two hydrogen bonds, namely N1–H···Cl2^i^ and N2–H···Cl2^ii^, and acts as a proton donor to two symmetry-related chloride counteranions as proton acceptors (Figure 10). Furthermore, a marked positive partial charge on the amide hydrogen is presumably responsible for the geometric nonsymmetry of the two H-bonds (see Table 6 and Figure 10). We can describe the first interaction as an ionic hydrogen bond [92]. Being predominantly Coulombic in nature, it remains directional, with N1–H pointing at chloride counteranion Cl2^i^. A similar interaction is well documented as salt-bridges between primary ammonium and carboxylate groups in biological systems [93].

**Table 6:** Hydrogen-bond geometry (Å, °) for **13**.

*D–*H···*A*	*D–*H	H···*A*	*D*···*A*	*D–*H···*A*
O1–H1···O2	0.84	1.771	2.5203(13)	147.4
N1–H1A···Cl2^i^	0.93	2.145	3.0617(11)	168.4
N2–H2···Cl2^ii^	0.88	2.360	3.2191(12)	165.4

Symmetry codes: (i) *x*, *y* + 1, *z*; (ii) −*x*, −*y* + 1, −*z* + 1.

#### 2.2.3. *Diethyl-[2-hydroxy-3-(3-methyl-butylcarbamoyl)-benzyl]-ammonium Chloride* (**14**)

**Figure 11:**
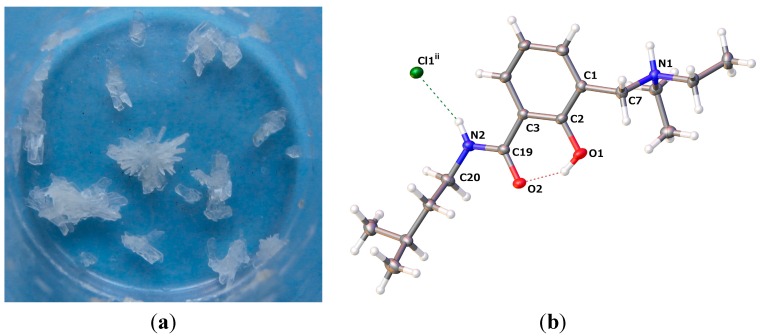
(**a**) Crystals grown for **14**; (**b**) molecular structure of **14** (drawn at a 50% probability level; dashed lines: hydrogen bonding).

The protonation of the Mannich base nitrogen in hybrid **3** triggers the same conformational switch from β- to α-form as shown for hybrid **9** (Section 2.2.2). The molecular structure of **14** (**3** ×HCl) is shown in Figure 11 and the selected geometrical parameters in Table 4.

**Figure 12:**
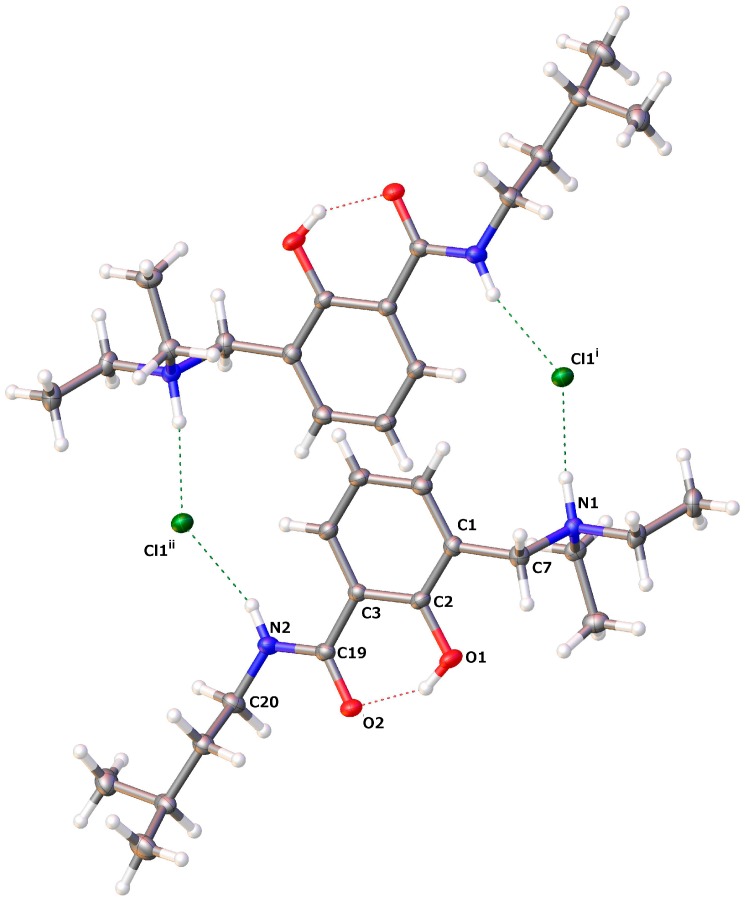
Crystal packing pattern showing intermolecular H-bonding to chloride ions in dimers of **14** (dashed lines: hydrogen bonding).

Furthermore, the crystals of **14** are composed of centrosymmetric dimers held by H-bonding interactions, which are nonsymmetric due to an additional Coulombic component in the interaction between the chloride anion and the ammonium cation. Like in **13**, each protonated molecule acts as a proton donor in two H-bonds, N1–H**···**Cl1^i^ and N2–H···Cl1^ii^ (Figure 12 and Table 7).

**Table 7:** Hydrogen-bond geometry (Å, °) for **14**.

*D–*H···*A*	*D–*H	H···*A*	*D*···*A*	*D–*H···*A*
O1–H1···O2	0.84	1.735	2.4905(11)	148.6
N1–H1A···Cl1^i^	0.93	2.158	3.075(9)	168.8
N2–H2···Cl1^ii^	0.88	2.399	3.2243(9)	156.4

Symmetry codes: (i) *x* + 1, −*y* + 1, −*z* + 1; (ii) *x* + ½, *y* − ½, *z*.

## 3. Experimental Section

### 3.1. Materials and Methods

Starting materials (amines, salicylic acid derivatives) were purchased from various commercial sources and were used without further purification. Solvents used in the synthesis and chromatographic purification steps were distilled prior use (ethyl acetate, petrol ether, *n*-hexane).

### 3.2. Reaction Monitoring and Purification of Compounds

Reaction monitoring was performed by thin layer chromatography (TLC) on Merck silica gel 60-F_254_ glass plates or on Macherey & Nagel POLYGRAM SIL G/UV 254 aluminum foils. The plates were developed with mixtures of hexane/ethyl acetate, neat ethyl acetate and methanol/ethyl acetate/aqueous ammonia. Compound spots were visualized by UV (254 nm) irradiation in a dual lamp CAMAG UV cabinet or in a TLC chamber containing iodine adsorbed on silica gel. Purification of compounds was performed by preparative separation by middle pressure liquid chromatography (MPLC) on silica gel 60 from Merck (0.040–0.063 μm, 240–400 mesh). Stationary phase material and the MPLC system consisting of unique home-built columns, a FMI pump (Fluid Metering, Inc., Syosset, Nassau County, NY, USA) and an Amersham Superfrac fraction collector were provided by H. Gstach from private ownership.

### 3.3. Analytical Characterization (mp, NMR)

Melting points (mp) were determined with a Bausch & Lomb microscope equipped with a Kofler melting stage and are uncorrected. NMR spectra were recorded on Bruker Avance 400-MHz and 600-MHz spectrometers (NMR Centre at the Faculty of Chemistry, University of Vienna). The software used for processing of 1D- (^1^H, ^13^C) and 2D- (COSY, HMBC, HSQC) NMR spectra was SpinWorks 3.1.7 (copyright 2010, Kirk Marat, University of Manitoba). Coupling constants (*J*) are given in Hertz (Hz) and refer to the first order interpretation (apparent coupling constants *J_a_*_pp_ are given). ^x^*J* refers to homonuclear HH coupling over x bonds. Solvents used for NMR spectroscopy: CDCl_3_, chloroform-*d*_1_ (CAS RN 865-49-6), was filtered through basic, activated aluminum oxide (Sigma Aldrich, St. Louis, MO, USA) prior to use; DMSO-d_6_, hexadeutero dimethyl sulfoxide (CAS RN 2206-27-1) was stored over a molecular sieve (4 Å). 2D NMR techniques used for the assignment of ^1^H and ^13^C resonance signals: HSQC (heteronuclear single quantum coherence), HMBC (heteronuclear multiple bond correlation) and COSY (correlation spectroscopy). Chemical shift calibration [94]: CDCl_3_, ^1^H δ = 7.26, ^13^C δ = 77.16; DMSO-d_6_, ^1^H δ = 2.50, ^13^C δ = 39.52 ppm.

### 3.4. Synthetic Procedures

#### 3.4.1. Syntheses of Starting Materials **1** and **5**

2-Hydroxy-*N-*(3-methyl-butyl)-benzamide (**1**) (CAS RN 24469-55-4) was prepared from 2-hydroxy-benzoic acid methyl ester following a literature procedure [95]. 5-chlorosalicylic acid amide **5** (CAS RN 1019327-19-5) was synthesized from 5-chloro-2-hydroxy-benzoic acid following a literature procedure [86]. The starting materials **1** and **5** were known, but NMR data in chloroform-*d*_1_ were not available in the primary literature (analytical data are provided in the Supporting Information).

#### 3.4.2. Aminomethylation of **1**: Separation of Isomers **2**–**4**

**Scheme 3:**
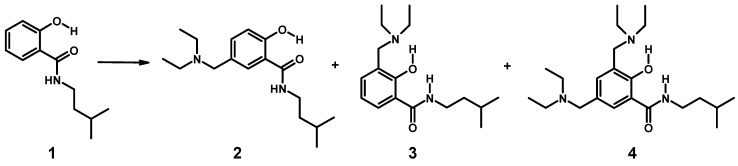
Synthesis of isomers **2**–**4** by aminomethylation of salicylamide **1**.

The synthesis of **2**–**4** by the Mannich reaction is depicted in Scheme 3.

To a solution of 2-hydroxy-*N-*(3-methyl-butyl)-benzamide (**1**) (4.03 g, 19.4 mmol) in ethanol (25 mL) was added diethyl amine (1.70 g, 1.2 equiv) and formaldehyde (1.89 g, ~37% in water). The mixture was transferred to a screw cap tube, sealed and kept in an oil bath at 90 °C for 20 h. The reaction mixture was cooled to room temperature. Volatile materials were removed under reduced pressure on a rotary evaporator. The oil obtained was stirred with hydrochloric acid (2 M, 20 mL) for 10 min. The acidic phase was extensively extracted with methyl *t*-butyl ether (MTBE) (4 × 25 mL). To the aqueous phase was added sodium hydroxide (2 M, 25 mL) and brine (25 mL). The basic products were extracted with ethyl acetate (2 × 30 mL). The organic phase was washed with water to pH 7 and dried over Na_2_SO_4_. After filtration, the solvent was removed on a rotary evaporator to give 2.9 g of residual material. The latter was subjected to MPLC-chromatography. Elution was started with MTBE (100%), and the fractions containing pure isomer **2** and **3** were collected. 5-Diethylaminomethyl-2-hydroxy-*N-*(3-methyl-butyl)-benzamide (**2**): 160 mg (3%, yellowish oil); 3-diethylaminomethyl-2-hydroxy-*N-*(3-methyl-butyl)-benzamide (**3**): 834 mg (15%, yellowish oil). The eluent was changed to a polar mixture made of ethyl acetate, methanol and aqueous ammonia (6/3.5/0.5). All residual material was eluted from the column: 966 mg, orange colored oil. The mixture was dissolved in MTBE (10 mL), and hydrogen chloride was added (1 mL, 4 M in dioxane). To the solution was added dry diethyl ether (20 mL). After cooling to 4 °C for 2 h, the solvents were decanted from the formed oil. The oil was washed with dry ether (3 × 10 mL). The basic products were liberated by the addition of NaHCO_3_ and extracted with ethyl acetate (80 mL). The organic phase was washed with brine, dried with sodium sulfate and evaporated. The residue was subjected to a second column chromatography applying ethyl acetate, methanol and aqueous ammonia at a ratio of 4/0.8/0.2 as the eluent. Fractions containing pure bis-aminomethyl substituted product were collected: 3,5-bis-diethylaminomethyl-2-hydroxy-*N-*(3-methyl-butyl)-benzamide (**4**): 190 mg (13%, slightly yellowish oil). Structural assignment of **2**–**4** was performed by NMR spectroscopy (Section 2.1.; the full assignment of NMR spectra is provided in the Supporting Information).

#### 3.4.3. Aminomethylation of 5-Chloro-2-hydroxy-(3-methyl-butyl)-benzamide (**5**)

The general procedure for the syntheses of compounds **6**–**11** (Scheme 2): A mixture of salicylamide **5** (1 mmol), formaldehyde (~37% in water) (1.2 mmol) and the corresponding secondary amine, **6**–**11** (1.2 mmol), in ethanol (4 mL) was prepared in a screw cap tube. The tube was sealed and kept in an oil bath at 90 °C for 20 h. The mixture obtained was diluted with water and extracted with ethyl acetate. The organic phase was washed once with sodium bicarbonate, two times with water and dried over magnesium sulfate. From the dried solution, the solvent was removed under reduced pressure on a rotary evaporator. The resulting residue was purified by column chromatography (silica gel, eluent: ethyl acetate/*n*-hexane).

#### 3.4.4. Synthesis of 5-Chloro-3-(1,3-dioxo-1,3-dihydro-isoindol-2-ylmethyl)-2-hydroxy-benzoic Acid (**12**)

The Tscherniac–Einhorn reaction is described for salicylic acids, but not for salicylamides [82]. A literature search for imidomethylation of salicylamides did not deliver any result. Only three simple salicylamides with the 3-phthalimidomethyl substituent were found (CAS RN: 1243458-02-7, 1243454-79-6, 1243288-23-4), but no references, experiments or characterization were available (commercial sources). Synthesis of compound **12** from **5** was performed by following Scheme 2.

Sulfuric acid (95%–98%, 40 mL) was cooled to 0 °C in an ice bath. To the cold acid was added 2-hydroxymethyl-isoindole-1,3-dione (CAS RN 118-29-6) [96] (1.77 g, 10 mmol) in one portion. The mixture was stirred for 10 min. To the turbid, colorless slurry was added **5** (2.42 g, 10 mmol). A clear solution formed after stirring for 10 min. The ice bath was removed, and stirring at room temperature was continued for 2 h. The color of the solution turned to a light yellow. The reaction was quenched by the addition of water (containing crushed ice, 150 mL). A colorless precipitate formed. The crude product was extracted with ethyl acetate (100 mL). The organic phase was washed with water until the water washings were neutral, dried over sodium sulfate and concentrated on a rotary evaporator. The product was purified by column chromatography. 5-Chloro-3-(1,3-dioxo-1,3-dihydro-isoindol-2-ylmethyl)-2-hydroxy-*N-*(3-methyl-butyl)-benzamide (**12**): (yield: 69%, mp 174–176 °C).

#### 3.4.5. Syntheses of Hydrochlorides **13**–**15** and Single Crystal Growth (Scheme 4)

The free base **9** (400 mg) was dissolved in MTBE (20 mL). To the solution was added 4 M hydrogen chloride in dioxane (1 mL) and dry diethyl ether (20 mL). A colorless oil formed. The solvents were decanted, and the oil was washed with diethyl ether (10 mL). Subsequently, the residual oil was dissolved in a mixture of ethyl acetate, a little methanol and MTBE. The solution obtained was transferred to an open Erlenmeyer flask. The solution concentrated slowly at room temperature. The hydrochloride of **9** started to crystallize. The colorless needles formed were collected by filtration and dried at room temperature: 1-[5-chloro-2-hydroxy-3-(3-methyl-butylcarbamoyl)-benzyl]-4-phenyl-piperidinium chloride (**13**); mp 234–237 °C.

**Scheme 4:**
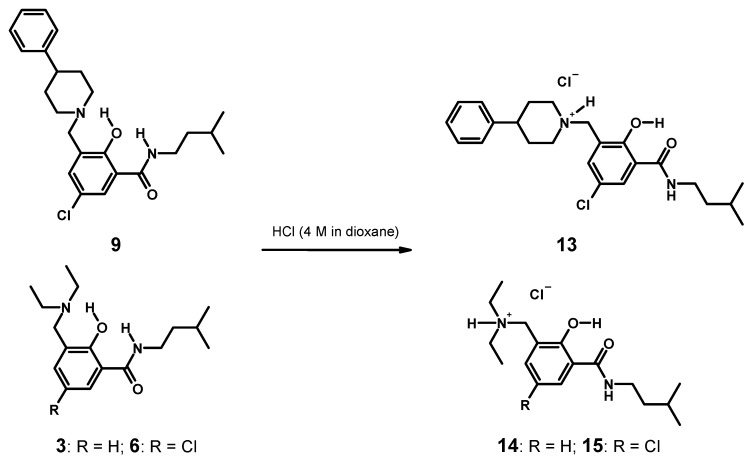
Syntheses of hydrochlorides **13**–**15**.

Derivative **3** (or **6**) (100 mg) was dissolved in MTBE (20 mL). To the solution was added 4 M hydrogen chloride in dioxane (1 mL) and dry diethyl ether (20 mL). The hydrochloride precipitated as oil. The solvents were decanted. The oily residue was heated in dry diethyl ether (10 mL). Crystallization started upon cooling in an ice bath. The ice bath was removed and the solution concentrated slowly at room temperature. The crystals were collected by filtration and washed once with cold diethyl ether. Diethyl-[2-hydroxy-3-(3-methyl-butyl-carbamoyl)-benzyl]-ammonium chloride (**14**): mp 172–174 °C. [5-Chloro-2-hydroxy-3-(3-methyl-butylcarbamoyl)-benzyl]-diethyl-ammonium chloride (**15**): mp 224–227 °C.

### 3.5. Crystallographic Structure Determination

X-ray diffraction measurements were performed on Bruker D8 VENTURE diffractometers. Single crystals were positioned at 50, 35, and 40 mm from the detector, and 2281, 2534, and 3016 frames were measured, each for 16, 5.6, and 24 s over a 0.4° scan width for **9**, **13**, and **14**, correspondingly. The data were processed using SAINT software [97]. Crystal data, data collection parameters and structure refinement details are given in Table 8. The structures were solved by direct methods and refined by full-matrix least-squares techniques. Non-H atoms were refined with anisotropic displacement parameters. H atoms were inserted in the calculated positions and refined with a riding model. The following computer programs were used: structure solution, *SHELXS-97*, and refinement, *SHELXL-97* [98]; molecular diagrams, *ORTEP* [99].

**Table 8:** Crystallographic data and refinement of compounds **9**, **13**, and **14**. (CCDC, Cambridge Crystallographic Data Centre).

	9	13	14
CCDC deposition number	1034222	1034223	1034221
Identification code	hugs157	hugs1572	hugs014
Empirical formula	C_24_H_31_ClN_2_O_2_	C_24_H_32_Cl_2_N_2_O_2_	C_17_H_29_ClN_2_O_2_
Formula weight	414.96	451.42	328.87
Temperature	100(2) K	100(2) K	100(2) K
Wavelength	0.71073 Å	0.71073 Å	0.71073 Å
Crystal system	monoclinic	triclinic	monoclinic
Space group	*P*2_1_*/n*	*P-*1	*C*2*/c*
Unit cell dimensions			
*a* (Å)	5.8621(3)	9.3714(4)	19.8617(17)
*b* (Å)	14.2609(7)	11.9179(5)	8.5447(6)
*c* (Å)	25.7776(12)	12.0435(5)	22.7687(19)
α (°)		108.9212(14)	
β (°)	92.9431(15)	100.3377(15)	110.078(5)
γ (°)		103.9072(14)	
Volume	2,152.13(18) Å^3^	1185.61(9) Å^3^	3629.3(5) Å^3^
Z	4	2	8
Density calculated	1.281 g/cm^3^	1.264 g/cm^3^	1.204 g/cm^3^
Absorption coefficient	0.200 mm^−1^	0.296 mm^−1^	0.220 mm^−1^
F(000)	888	480	1424
Crystal size (mm)	0.23 × 0.18 × 0.08	0.21 × 0.21 × 0.13	0.30 × 0.14 × 0.06
*θ* range for data collection	2.13–30.04°	1.86–30.12°	1.90–30.03°
Index ranges	−8 ≤ h ≤ 8	−13 ≤ h ≤ 13	−27 ≤ h ≤ 27
	−20 ≤ k ≤ 20	−16 ≤ k ≤ 16	−12 ≤ k ≤ 12
	−36 ≤ l ≤ 36	−16 ≤ l ≤ 16	−31 ≤ l ≤ 32
Reflections collected	60,472	54,620	84,947
Reflections independent	6292 [R_int_] = 0.0290	6974 [R_int_] = 0.0433	5299 [R_int_] = 0.0377
Completeness to 2*θ* = 30.04	100%	99.9%	99.9%
Transmission *T_max_/T_min_*	0.9847/0.9561	0.9617/0.9399	0.9870/0.9371
Refinement method	Full-matrix least-squares on F^2^		
Data/restraints/parameters	6292/0/265	6974/0/274	5299/0/204
Goodness-of-fit on F^2^	1.012	1.006	1.027
Final R indices (*I > 2σ(I)*)	*R1* (obs. data) = 0.0349	*R1* (obs. data) = 0.0387	*R1* (obs. data) = 0.0351
*R* indices (all data)	*wR2* = 0.0957	*wR2* = 0.1084	*wR2* = 0.0967
Largest diff. Peak and hole	0.455 and −0.201 e.Å^3^	1.035 and −0.295 e.Å^3^	0.456 and −0.264 e.Å^3^

CCDC 1034221, CCDC 1034222, and CCDC 1034223 contain the supplementary crystallographic data for this paper. These data can be obtained free of charge via http://www.ccdc.cam.ac.uk/conts/retrieving.html (or from the CCDC, 12 Union Road, Cambridge CB2 1EZ, UK; Fax: +44 1223 336033; E-mail: deposit@ccdc.cam.ac.uk).

## 4. Conclusions

Salicylalkylamides undergo a conformational switch from the α- to the β-form upon aminomethylation in the 3-position. The reaction creates a hybrid composed of salicylamide and a Mannich base. The conformational change can be reversed by *N*-protection or protonation of the Mannich base nitrogen. The preferred β-conformation of the hybrid molecules adopted in solution has been determined by NMR spectroscopy. Single crystal X-ray diffraction revealed the β-form for hybrids of salicylalkylamide and the Mannich base, also in the solid state. The reversal of the conformation upon protonation has been demonstrated for the solid state by the X-ray diffraction data of the hydrochlorides of hybrid molecules. The structural changes observed upon transformation of salicylalkylamide-Mannich base hybrids from the α- to the β-conformation provide powerful tools for the design of more complex molecular architectures, as well as of novel drugs. Decorating salicylamides with a Mannich base motif in the 3-position creates an extended pharmacophore whose conformation can be controlled. Hybrid molecules in the α-form can cover more extended interactions with targets, whereas the β-form provides an L-shape conformation able to fill steep pockets, e.g., in a protein. The two amine diversity sites, one in the Mannich base, the other in the amide part of the hybrid, can be addressed independently by the design. In addition, double intramolecular hydrogen bonding in the β-form of hybrid molecules will influence the pharmacological properties by masking the polarity of the functional groups involved.

## Supplementary Materials

Supplementary materials can be accessed at: http://www.mdpi.com/1420-3049/20/01/1686/s1.
