# Supplementary material for: Hybrids of Salicylalkylamides and Mannich Bases: Control of the Amide Conformation by Hydrogen Bonding in Solution and in the Solid State
Source: Molecules. 2015 Jan 20;20(1):1686–711. doi: 10.3390/molecules20011686 (PMC6272445; doi:10.3390/molecules20011686)

Supporting Information

Content:

1. List of Compounds (Assignment/NMR Spectra)
2. Resonance-Assisted Hydrogen Bonding, Resonance Structures: Figure S1
3. Analytical Data and Assignment of the ^{1}H/^{13}C Resonances of Compounds **1**–**15**
4. Representation of the ^{1}H/^{13}C NMR Spectra of Compounds **1**–**15**
5. Crystals Grown for Free Base **9** and Hydrochlorides **13** and **14**

S1. List of Compounds (Assignment/NMR Spectra)

(**1**) 2-Hydroxy-*N*-(3-methyl-butyl)-benzamide
(**2**) 5-Diethylaminomethyl-2-hydroxy-*N*-(3-methyl-butyl)-benzamide
(**3**) 3-Diethylaminomethyl-2-hydroxy-*N*-(3-methyl-butyl)-benzamide
(**4**) 3,5-Bis-diethylaminomethyl-2-hydroxy-*N*-(3-methyl-butyl)-benzamide
(**5**) 5-Chloro-2-hydroxy-*N*-(3-methyl-butyl)-benzamide
(**6**) 5-Chloro-3-diethylaminomethyl-2-hydroxy-*N*-(3-methyl-butyl)-benzamide
(**7**) 5-Chloro-3-dibutylaminomethyl-2-hydroxy-*N*-(3-methyl-butyl)-benzamide
(**8**) 5-Chloro-2-hydroxy-*N*-(3-methyl-butyl)-3-piperidin-1-ylmethyl-benzamide
(**9**) 5-Chloro-2-hydroxy-*N*-(3-methyl-butyl)-3-(4-phenyl-piperidin-1-ylmethyl)-benzamide
(**10**) 3-(4-Benzyl-piperidin-1-ylmethyl)-5-chloro-2-hydroxy-*N*-(3-methyl-butyl)-benzamide
(**11**) 5-Chloro-2-hydroxy-*N*-(3-methyl-butyl)-3-(4-phenyl-piperazin-1-ylmethyl)-benzamide
(**12**) 5-Chloro-3-(1,3-dioxo-1,3-dihydro-isoindol-2-ylmethyl)-2-hydroxy-*N*-(3-methyl-butyl)-benzamide
(**13**) 1-[5-Chloro-2-hydroxy-3-(3-methyl-butylcarbamoyl)-benzyl]-4-phenyl-piperidinium chloride
(**14**) Diethyl-[2-hydroxy-3-(3-methyl-butylcarbamoyl)-benzyl]-ammonium chloride
(**15**) [5-Chloro-2-hydroxy-3-(3-methyl-butylcarbamoyl)-benzyl]-diethyl-ammonium chloride

S2. Resonance-Assisted Hydrogen Bonding, Resonance Structures: Figure S1

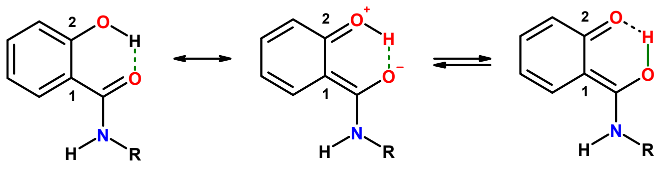

Resonance structures of α - form showing the quinoid forms for *C*-1 and *C*-2

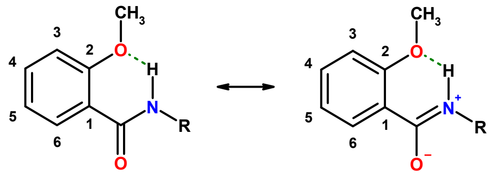

Resonance structures of β - form showing N lone pair delocalization into the amide carbonyl

Figure S1. Selected resonance structures for the α- and the β-form of salicylamides (dashed lines: hydrogen bonding).

S3. Analytical Data and Assignment of the ^{1}H/^{13}C Resonances of Compounds 1–15

Abbreviations: br, broad; dyn, dynamic; salicyl, aromatic ring of the salicylic acid substructure; multiplicity: m, multiplet; s, singlet, d, doublet; t, triplet; q, quaternary or quartet; qu, quintet; sept, septet; sext, sextet; AA' or BB', part of an AA'BB' spin system; m [t, dd, q, tt, sext], the notation in brackets describes the overall appearance of the signal pattern (e.g., m[d] represents a multiplet with the appearance like a doublet); ax, axial; eq, equatorial.

(1) 2-Hydroxy-*N*-(3-methyl-butyl)-benzamide

^{1}H-NMR (400 MHz, $CDCl_3$, 23 °C): δ = 0.94 (d, ^{3}J = 6.6, 6H, CONHCH$_2$CH$_2$CH(C*H*$_3$)$_2$), 1.47–1.54 (m, 2H, CONHCH$_2$C*H*$_2$CH(CH$_3$)$_2$), 1.67 (sept, ^{3}J = 6.6, 1H, CONHCH$_2$CH$_2$C*H*(CH$_3$)$_2$), 3.42–3.49 (m, 2H, CONHC*H*$_2$CH$_2$CH(CH$_3$)$_2$), 6.41 (s, br, 1H, CON*H*), 6.79–6.85 (m[t], 1H, *H*-5 salicyl), 6.96 (d, ^{3}J = 8.1, 1H, *H*-3 salicyl), 7.35 (d, ^{3}J = 8.1, 1H, *H*-6 salicyl), 7.36 (overlay with *H*-6, m[tr], *H*-4 salicyl), 12.43 (sharp, 1H, salicyl-2-O*H*).

^{13}C{^{1}H}NMR (100 MHz, $CDCl_3$, 23 °C): δ = 22.53 (NCH$_2$CH$_2$CH(*C*H$_3$)$_2$), 26.05 (NCH$_2$CH$_2$-*C*H(CH$_3$)$_2$), 38.14 (N*C*H$_2$CH$_2$CH(CH$_3$)$_2$), 38.40 (NCH$_2$*C*H$_2$CH(CH$_3$)$_2$), 114.53 (*C*$_q$-1 salicyl), 118.66, 118.73 (*C*-3, *C*-5 salicyl), 125.41 (*C*-6 salicyl), 134.18 (*C*-4 salicyl), 161.58 (*C*$_q$-2 salicyl), 170.04 (*C*ONH).

(2) 5-Diethylaminomethyl-2-hydroxy-*N*-(3-methyl-butyl)-benzamide

Yellowish oil, yield: 3%.

^{1}H-NMR (600 MHz, $CDCl_3$, 23 °C): δ = 0.95 (d, ^{3}J = 6.6, 6H, CONHCH$_2$CH$_2$CH(C*H*$_3$)$_2$), 1.03 (t, ^{3}J = 7.1, 6H, N(CH$_2$C*H*$_3$)$_2$), 1.49–1.54 (m, 2H, CONHCH$_2$C*H*$_2$CH(CH$_3$)$_2$), 1.68 (sept, ^{3}J = 6.6, 1H, CONHCH$_2$CH$_2$C*H*(CH$_3$)$_2$), 2.52 (q, ^{3}J = 7.1, 4H, N(C*H*$_2$CH$_3$)$_2$), 3.42–3.46 (m, 2H, CONHC*H*$_2$CH$_2$-CH(CH$_3$)$_2$), 3.47 (s, 2H, salicyl-5-C*H*$_2$N(CH$_2$CH$_3$)$_2$), 6.53 (s, br, 1H, CON*H*CH$_2$CH$_2$CH(CH$_3$)$_2$), 6.89 (d, ^{3}J = 8.5, 1H, *H*-3 salicyl), 7.29 (dd, ^{3}J = 8.4, ^{4}J = 1.9, 1H, *H*-4 salicyl), 7.38 (d, 1H, ^{4}J = 1.6, 1H, *H*-6 salicyl), salicyl-2-O*H* in rapid exchange.

^{13}C{^{1}H}NMR (125 MHz, $CDCl_3$, 23 °C): δ = 11.47 (CH$_2$N(CH$_2$*C*H$_3$)$_2$), 22.59 (NCH$_2$CH$_2$-CH(*C*H$_3$)$_2$), 26.10 (NCH$_2$CH$_2$*C*H(CH$_3$)$_2$), 38.16 (N*C*H$_2$CH$_2$CH(CH$_3$)$_2$), 38.43 (NCH$_2$*C*H$_2$CH(CH$_3$)$_2$), 46.58 (CH$_2$N(*C*H$_2$CH$_3$)$_2$), 57.11 (salicyl-5-*C*H$_2$N(CH$_2$CH$_3$)$_2$), 114.43 (*C*$_q$-1 salicyl), 118.19 (*C*-3 salicyl), 125.77 (*C*-6 salicyl), 129.63 (*C*$_q$-5 salicyl), 134.95 (*C*-4 salicyl), 160.53 (*C*$_q$-2 salicyl), 170.04 (*C*ONHCH$_2$CH$_2$CH(CH$_3$)$_2$).

(3) 3-Diethylaminomethyl-2-hydroxy-*N*-(3-methyl-butyl)-benzamide

Yellowish oil, yield: 15%.

^{1}H-NMR (600 MHz, $CDCl_3$, 23 °C): δ = 0.95 (d, ^{3}J = 6.6, 6H, CONHCH$_2$CH$_2$CH(C*H*$_3$)$_2$), 1.14 (t, ^{3}J = 7.1, 6H, N(CH$_2$C*H*$_3$)$_2$), 1.50–1.54 (m, 2H, CONHCH$_2$C*H*$_2$CH(CH$_3$)$_2$), 1.69 (sept, ^{3}J = 6.6, 1H, CONHCH$_2$CH$_2$C*H*(CH$_3$)$_2$), 2.68 (q, ^{3}J = 7.1, 4H, N(C*H*$_2$CH$_3$)$_2$), 3.45–3.49 (m, 2H, CONHC*H*$_2$CH$_2$-CH(CH$_3$)$_2$), 3.82 (s, 2H, salicyl-3-C*H*$_2$N(CH$_2$CH$_3$)$_2$), 6.82 (dd[t], 2 × ^{3}J = 7.6, 1H, *H*-5 salicyl), 7.05 (d, ^{3}J = 6.9, 1H, *H*-4 salicyl), 8.08 (dd, ^{3}J = 7.9, ^{4}J = 1.8, 1H, *H*-6 salicyl), 8.56 (s, br, 1H, CON*H*), 7.50–9.50 (br, 1H, salicyl-2-O*H*).

^{13}C{^{1}H}NMR (125 MHz, CDCl$_3$, 23 °C): δ = 10.95 (CH$_2$N(CH$_2$*C*H$_3$)$_2$), 22.69 (NCH$_2$CH$_2$-CH(*C*H$_3$)$_2$), 26.24 (NCH$_2$CH$_2$*C*H(CH$_3$)$_2$), 38.03 (N*C*H$_2$CH$_2$CH(CH$_3$)$_2$), 38.71 (NCH$_2$*C*H$_2$CH(CH$_3$)$_2$), 46.09 (CH$_2$N(*C*H$_2$CH$_3$)$_2$), 56.85 (salicyl-3-*C*H$_2$N(CH$_2$CH$_3$)$_2$), 118.68 (*C*-5 salicyl), 119.91 (*C*$_q$-1 salicyl), 121.96 (*C*$_q$-3 salicyl), 130.78 (*C*-6 salicyl), 131.30 (*C*-4 salicyl), 158.18 (*C*$_q$-2 salicyl), 166.06 (*C*ONH).

(4) 3,5-Bis-diethylaminomethyl-2-hydroxy-*N*-(3-methyl-butyl)-benzamide

Yellowish oil, yield: 13%.

^{1}H-NMR (400 MHz, CDCl$_3$, 23 °C): δ = 0.91 (d, 6H, CONHCH$_2$CH$_2$CH(C*H*$_3$)$_2$), 0.98 (t, 6H, salicyl-5-CH$_2$N(CH$_2$C*H*$_3$)$_2$), 1.09 (t, 6H, salicyl-3-CH$_2$N(CH$_2$C*H*$_3$)$_2$), 1.46–1.51 (m, 2H, CONHCH$_2$-C*H*$_2$CH(CH$_3$)$_2$), 1.66 (m[sept], 1H, CONHCH$_2$CH$_2$C*H*(CH$_3$)$_2$), 2.46 (q, 4H, salicyl-5-CH$_2$N-(C*H*$_2$CH$_3$)$_2$), 2.62 (q, 4H, salicyl-3-CH$_2$N(C*H*$_2$CH$_3$)$_2$), 3.41–3.46 (m, 4H, CONHC*H*$_2$CH$_2$CH(CH$_3$)$_2$, salicyl-5-C*H*$_2$N(CH$_2$CH$_3$)$_2$), 3.78 (s, 2H, salicyl-3-C*H*$_2$N(CH$_2$CH$_3$)$_2$), 7.07 (d, ^4J = 2.8, 1H, *H*-4 salicyl), 7.90 (d, ^4J = 2.8, 1H, *H*-6 salicyl), 8.54 (br[t], 1H, CON*H*CH$_2$CH$_2$CH(CH$_3$)$_2$), 11.03 (br, 1H, salicyl-2-O*H*).

^{13}C{^{1}H}NMR (100 MHz, CDCl$_3$, 23 °C): δ = 10.93 (salicyl-3-CH$_2$N(CH$_2$*C*H$_3$)$_2$), 11.60 (salicyl-5-CH$_2$N(CH$_2$*C*H$_3$)$_2$), 22.58 (NCH$_2$CH$_2$CH(*C*H$_3$)$_2$), 26.11 (NCH$_2$CH$_2$*C*H(CH$_3$)$_2$), 37.90 (N*C*H$_2$CH$_2$-CH(CH$_3$)$_2$), 38.65 (NCH$_2$*C*H$_2$CH(CH$_3$)$_2$), 46.08 (salicyl-3-CH$_2$-N(*C*H$_2$CH$_3$)$_2$), 46.53 (salicyl-5-CH$_2$N-(*C*H$_2$CH$_3$)$_2$), 56.71 (salicyl-5-*C*H$_2$N(CH$_2$CH$_3$)$_2$), 56.83 (salicyl-3-*C*H$_2$N(CH$_2$CH$_3$)$_2$), 119.02 (*C*$_q$-1 salicyl), 122.05 (*C*$_q$-3 salicyl), 129.54 (*C*$_q$-5 salicyl), 130.98 (*C*-6 salicyl), 131.95 (*C*-4 salicyl), 156.93 (*C*$_q$-2 salicyl), 166.06 (*C*ONHCH$_2$CH$_2$CH(CH$_3$)$_2$).

(5) 5-Chloro-2-hydroxy-*N*-(3-methyl-butyl)-benzamide

Colorless crystals, yield: 78%, mp 70–72 °C.

^{1}H-NMR (400 MHz, CDCl$_3$, 23 °C): δ = 0.96 (d, 6H, CONHCH$_2$CH$_2$CH(C*H*$_3$)$_2$), 1.50–1.55 (m, 2H, CONHCH$_2$C*H*$_2$CH(CH$_3$)$_2$), 1.68 (m[sept], 1H, CONHCH$_2$CH$_2$C*H*(CH$_3$)$_2$), 3.44–3.49 (m, 2H, CONH-C*H*$_2$CH$_2$CH(CH$_3$)$_2$), 6.23 (br, s, 1H, N*H*), 6.93 (d, ^3J = 8.7, 1H, *H*-3 salicyl), 7.23 (d, 1H, *H*-6 salicyl), 7.25 (dd, 1H, *H*-4 salicyl), 12.29 (s, br, 1H, salicyl-2-O*H*).

^{13}C{^{1}H}NMR (100 MHz, CDCl$_3$, 23 °C): δ = 22.55 (NCH$_2$CH$_2$CH(*C*H$_3$)$_2$), 26.05 (NCH$_2$CH$_2$-*C*H(CH$_3$)$_2$), 38.34, 38.37 (N*C*H$_2$*C*H$_2$CH(CH$_3$)$_2$), 115.46 (*C*$_q$-1 salicyl), 120.29 (*C*-3 salicyl), 123.39 (*C*$_q$-5 salicyl), 125.01 (*C*-6 salicyl), 134.10 (*C*-4 salicyl), 160.22 (*C*$_q$-2 salicyl), 168.96 (*C*ONH).

(6) 5-Chloro-3-diethylaminomethyl-2-hydroxy-*N*-(3-methyl-butyl)-benzamide

Yellowish oil, yield: 24%.

^{1}H-NMR (400 MHz, CDCl$_3$, 23 °C): δ = 0.93 (d, 6H, CONHCH$_2$CH$_2$CH(C*H*$_3$)$_2$), 1.13 (t, 6H, N(CH$_2$C*H*$_3$)$_2$), 1.47–1.54 (m, 2H, CONHCH$_2$C*H*$_2$CH(CH$_3$)$_2$), 1.68 (m[sept], 1H, CONHCH$_2$CH$_2$-C*H*(CH$_3$)$_2$), 2.67 (q, 4H, N(C*H*$_2$CH$_3$)$_2$), 3.42–3.48 (m, 2H, CONHC*H*$_2$CH$_2$CH(CH$_3$)$_2$), 3.78 (s, 2H, C*H*$_2$N(CH$_2$CH$_3$)$_2$), 6.99 (d, ^4J = 2.8, 1H, *H*-4 salicyl), 8.03 (d, ^4J = 2.8, 1H, *H*-6 salicyl), 8.55 (br[t], 1H, CON*H*CH$_2$CH$_2$CH(CH$_3$)$_2$), 11.16 (br, 1H, salicyl-2-O*H*).

^{13}C{^{1}H}NMR (100 MHz, CDCl$_3$, 23 °C): δ = 10.84 (CH$_2$N(CH$_2$*C*H$_3$)$_2$), 22.64 (NCH$_2$CH$_2$-CH(*C*H$_3$)$_2$), 26.19 (NCH$_2$CH$_2$*C*H(CH$_3$)$_2$), 38.10 (N*C*H$_2$CH$_2$CH(CH$_3$)$_2$), 38.62 (NCH$_2$*C*H$_2$CH(CH$_3$)$_2$), 46.16

($CH_2N(\mathit{C}H_2CH_3)_2$), 56.47 ($\mathit{C}H_2N(CH_2CH_3)_2$), 121.30 ($\mathit{C}_q$-1 salicyl), 123.41 ($\mathit{C}_q$-5 salicyl), 123.56 ($\mathit{C}_q$-3 salicyl), 130.19 (*C*-6 salicyl), 130.76 (*C*-4 salicyl), 157.09 (C_q-2 salicyl), 164.82 (*C*ONH).

(7) 5-Chloro-3-dibutylaminomethyl-2-hydroxy-*N*-(3-methyl-butyl)-benzamide

Orange oil, yield: 51%.

^{1}H-NMR (400 MHz, $CDCl_3$, 23 °C): δ = 0.91 (t, ^3J = 7.4, 6H, $N(CH_2CH_2CH_2C\mathit{H}_3)_2$), 0.93 (d, ^3J = 8.1, 6H, $CONHCH_2CH_2CH(C\mathit{H}_3)_2$), 1.25–1.36 (m, 4H, $N(CH_2CH_2C\mathit{H}_2CH_3)_2$), 1.46–1.57 (m, 6H, $CONHCH_2C\mathit{H}_2CH(CH_3)_2$, $N(CH_2C\mathit{H}_2CH_2CH_3)_2$), 1.63–1.74 (m[sept], 1H, $CONHCH_2CH_2$-$C\mathit{H}(CH_3)_2$), 2.52–2.59 (m[t], 4H, $N(C\mathit{H}_2CH_2CH_2CH_3)_2$), 3.42–3.49 (m, 2H, $CONHC\mathit{H}_2CH_2$-$CH(CH_3)_2$), 3.78 (s, 2H, salicyl-3-$C\mathit{H}_2N$(di-*n*-butyl), 6.99 (d, ^4J = 2.7, 1H, *H*-4 salicyl), 8.04 (d, ^4J = 2.7, 1H, *H*-6 salicyl), 8.57 (t, br, ^3J = 4.6, 1H, $CON\mathit{H}CH_2CH_2CH(CH_3)_2$), 11.03 (br, 1H, salicyl-2-O*H*).

^{13}C{^{1}H}NMR (100 MHz, $CDCl_3$, 23 °C): δ = 13.95 (*C*-4 of $N(CH_2CH_2CH_2\mathit{C}H_3)_2$), 20.54 (*C*-3 of $N(CH_2CH_2\mathit{C}H_2CH_3)_2$), 22.62 ($CONHCH_2CH_2CH(\mathit{C}H_3)_2$), 26.15 ($CONHCH_2CH_2\mathit{C}H(CH_3)_2$), 27.96 (*C*-2 of $N(CH_2\mathit{C}H_2CH_2CH_3)_2$), 38.03 ($CONH\mathit{C}H_2CH_2CH(CH_3)_2$), 38.55 ($CONHCH_2\mathit{C}H_2CH(CH_3)_2$), 52.83 (*C*-1 of $N(\mathit{C}H_2CH_2CH_2CH_3)_2$), 57.70 (salicyl-3-$\mathit{C}H_2N$(di-*n*-butyl), 121.28 ($\mathit{C}_q$-1 salicyl), 123.43 ($\mathit{C}_q$-5 salicyl), 123.73 ($\mathit{C}_q$-3 salicyl), 130.21 (*C*-6 salicyl), 130.78 (*C*-4 salicyl), 156.97 (C_q-2 salicyl), 164.84 ($\mathit{C}ONHCH_2CH_2CH(CH_3)_2$).

(8) 5-Chloro-2-hydroxy-*N*-(3-methyl-butyl)-3-piperidin-1-ylmethyl-benzamide

Yellowish crystals, yield: 58%, mp: 90–91 °C.

^{1}H-NMR (400 MHz, $CDCl_3$, 23 °C): δ = 0.95 (d, ^3J = 6.5, 6H, $CONHCH_2CH_2CH(C\mathit{H}_3)_2$), 1.47–1.74 (m, 9H, $C\mathit{H}_2$-3,4,5 piperidine, $CONHCH_2C\mathit{H}_2C\mathit{H}(CH_3)_2$), 2.03–3.18 (br, dyn, 4H, $C\mathit{H}_2$-2,6 piperidine), 3.42–3.50 (m, 2H, $CONHC\mathit{H}_2CH_2CH(CH_3)_2$), 3.69 (s, 2H, salicyl-3-$C\mathit{H}_2N$(piperidine)), 7.01 (d, ^4J = 2.5, 1H, *H*-4 salicyl), 8.04 (d, ^4J = 2.7, 1H, *H*-6 salicyl), 8.47 (br[t], 1H, $CON\mathit{H}CH_2CH_2CH(CH_3)_2$), 10.18 (s, br, 1H, salicyl-2-O*H*).

^{13}C{^{1}H}NMR (100 MHz, $CDCl_3$, 23 °C): δ = 22.67 ($NCH_2CH_2CH(\mathit{C}H_3)_2$), 23.69 ($\mathit{C}H_2$-4 piperidine), 25.61 ($\mathit{C}H_2$-3,5 piperidine), 26.23 ($NCH_2CH_2\mathit{C}H(CH_3)_2$), 38.16 ($N\mathit{C}H_2CH_2CH(CH_3)_2$), 38.65 ($NCH_2\mathit{C}H_2CH(CH_3)_2$), 53.59 (*C*-2,6 piperidine), 61.44 (salicyl-3-$\mathit{C}H_2N$(piperidine)), 121.24 (C_q-1 salicyl), 123.22 (C_q-3 salicyl), 123.70 (C_q-5 salicyl), 130.25 (*C*-6 salicyl), 131.04 (*C*-4 salicyl), 156.63 (C_q-2 salicyl), 164.81 (*C*ONH).

(9) 5-Chloro-2-hydroxy-*N*-(3-methyl-butyl)-3-(4-phenyl-piperidin-1-ylmethyl)-benzamide

Yellowish crystals, yield: 67%, mp: 130–131 °C.

^{1}H-NMR (400 MHz, $CDCl_3$, 23 °C): δ = 0.98 (d, ^3J = 6.5, 6H, $CONHCH_2CH_2CH(C\mathit{H}_3)_2$), 1.52–1.60 (m, 2H, $CONHCH_2C\mathit{H}_2CH(CH_3)_2$), 1.67–1.77 (m[sept], 1H, $CONHCH_2CH_2C\mathit{H}(CH_3)_2$), 1.77–1.90 (m[ddd], 2H, $C\mathit{H}_{ax}$-3,5 piperidine), 1.94–2.02 (m[d], 2H, $C\mathit{H}_{eq}$-3,5 piperidine), 2.29–2.39 (m[t], 2H, $C\mathit{H}_{ax}$-2,6 piperidine), 2.58–2.69 (m[86], 1H, $C\mathit{H}_{ax}$-4 piperidine), 3.12–3.19 (m[d], 2H, $C\mathit{H}_{eq}$-2,6 piperidine), 3.47–3.54 (m, 2H, $CONHC\mathit{H}_2CH_2CH(CH_3)_2$), 3.78 (s, 2H, salicyl-3-$C\mathit{H}_2N$-piperidine), 7.07 (d, ^4J = 2.6, 1H, *H*-4 salicyl), 7.22–7.28 (m, 3H, *C*-2,6,4 phenyl in 4-phenylpiperidine), 7.31–7.37 (m,

2H, *C*-3,5 phenyl in 4-phenylpiperidine), 8.09 (d, ${}^4J$ = 2.7, 1H, *H*-6 salicyl), 8.44 (t, ${}^3J$ = 4.5, 1H, CON*H*$CH_2CH_2CH(CH_3)_2$), 11.59 (br, 1H, salicyl-2-O*H*).

${}^{13}C\{{}^1H\}$NMR (100 MHz, $CDCl_3$, 23 °C): δ = 22.64 (CONHCH_2CH_2CH(*C*$H_3)_2$), 26.20 (CONH-CH_2CH_2*C*$H(CH_3)_2$), 33.04 (*C*-3,5 piperidine), 38.17 (CONHCH_2*C*$H_2CH(CH_3)_2$), 38.61 (CONH-*C*$H_2CH_2CH(CH_3)_2$), 41.80 (*C*-4 piperidine), 53.44 (*C*-2,6 piperidine), 61.06 (salicyl-3-*C*H_2N-piperidine), 121.27 (C_q-1 salicyl), 123.39 (C_q-3 salicyl), 123.94 (C_q-5 salicyl), 126.66 (*C*-4 phenyl in 4-phenylpiperidine), 126.75 (*C*-2,6 phenyl in 4-phenylpiperidine), 128.66 (*C*-3,5 phenyl in 4-phenylpiperidine), 130.26 (*C*-6 salicyl), 131.05 (*C*-4 salicyl), 144.95 (C_q-1 phenyl in 4-phenyl-piperidine), 156.20 (C_q-2 salicyl), 164.65 (*C*ONH$CH_2CH_2CH(CH_3)_2$).

(10) 3-(4-Benzyl-piperidin-1-ylmethyl)-5-chloro-2-hydroxy-*N*-(3-methyl-butyl)-benzamide

Yellowish crystals, yield: 23%, mp: 100–101 °C.

${}^1H$-NMR (400 MHz, $CDCl_3$, 23 °C): δ = 0.96 (d, ${}^3J$ = 6.5, 6H, CONHCH_2CH_2CH(C*H*$_3)_2$), 1.29–1.41 (m[ddd], dyn, 2H, CH_{ax}-3,5 piperidine), 1.49–1.57 (m, 2H, CONHCH_2C*H*$_2CH(CH_3)_2$), 1.58–1.78 (m, 4H, CH_{eq}-3,5 piperidine, CONHCH_2CH_2C*H*$(CH_3)_2$, CH_{ax}-4 piperidine), 2.06–2.20 (m[t], dyn, 2H, CH_{ax}-2,6 piperidine), 2.57 (d, ${}^3J$ = 7.1, 2H, piperidine-4-C*H*$_2$-phenyl), 2.93–3.03 (m[d], 2H, CH_{eq} 2,6 piperidine), 3.43–3.51 (m, 2H, CONHC*H*$_2CH_2CH(CH_3)_2$), 3.68 (s, 2H, salicyl-3-C*H*$_2$-4-benzyl-piperidine), 6.99 (d, ${}^4J$ = 2.6, 1H, *H*-4 salicyl), 7.10–7.16 (m, 2H, *H*-2,6 phenyl in 4-benzylpiperidine), 7.17–7.23 (m, 1H, *H*-4 phenyl in 4-benzylpiperidine), 7.24–7.31 (m, 2H, *H*-3,5 phenyl in 4-benzylpiperidine), 8.05 (d, ${}^4J$ = 2.7, 1H, *H*-6 salicyl), 8.48 (t, ${}^3J$ = 5.0, 1H, CON*H*$CH_2CH_2CH(CH_3)_2$), 11.69 (s, br, 1H, salicyl-2-O*H*).

${}^{13}C\{{}^1H\}$NMR (100 MHz, $CDCl_3$, 23 °C): δ = 22.63 (CONHCH_2CH_2CH(*C*$H_3)_2$), 26.17 (CONH-CH_2CH_2*C*$H(CH_3)_2$), 31.78 (*C*-3,5 piperidine), 37.41 (*C*-4 4-benzylpiperidine), 38.13 (CONH*C*H_2CH_2-$CH(CH_3)_2$), 38.60 (CONHCH_2*C*$H_2CH(CH_3)_2$), 42.79 (4-piperidine-*C*H_2-phenyl), 52.93 (*C*-2,6 piperidine), 60.96 (salicyl-3-*C*H_2-1-piperidine-4-benzyl), 121.16 (C_q-1 salicyl), 123.29 (C_q-3 salicyl), 123.67 (C_q-5 salicyl), 126.15 (*C*-4 phenyl in 4-benzylpiperidine), 128.39 (*C*-3,5 phenyl in 4-benzylpiperidine), 129.09 (*C*-2,6 phenyl in 4-benzylpiperidine), 130.16 (*C*-6 salicyl), 130.93 (*C*-4 salicyl), 139.96 (C_q-1 phenyl in 4-benzylpiperidine), 156.44 (C_q-2 salicyl), 164.69 (*C*ONH$CH_2CH_2CH(CH_3)_2$).

(11) 5-Chloro-2-hydroxy-*N*-(3-methyl-butyl)-3-(4-phenyl-piperazin-1-ylmethyl)-benzamide

Colorless crystals, yield: 41%, mp: 100–101 °C.

${}^1H$-NMR (400 MHz, $CDCl_3$, 23 °C): δ = 0.94 (d, 6H, CONHCH_2CH_2CH(C*H*$_3)_2$), 1.48–1.55 (m, 2H, CONHCH_2C*H*$_2CH(CH_3)_2$), 1.63–1.74 (m[sept], 1H, CONHCH_2CH_2C*H*$(CH_3)_2$), 2.77 (m, br, 4H, C*H*$_2$-3,5 piperazine), 3.27 (m, br, 4H, C*H*$_2$-2,6 piperazine), 3.43–3.51 (m, 2H, CONHC*H*$_2CH_2CH(CH_3)_2$), 3.77 (s, 2H, salicyl-3-C*H*$_2$-1-piperazine-4-phenyl), 6.87–6.95 (m, 3H, *H*-2,4,6 phenyl), 7.09 (d, ${}^4J$ = 2.6, 1H, *H*-4 salicyl), 7.25–7.31 (m, 2H, *H*-3,5 phenyl), 8.06 (d, ${}^4J$ = 2.6, 1H, *H*-6 salicyl), 8.25 (t, ${}^3J$ = 5.0, 1H, CON*H*$CH_2CH_2CH(CH_3)_2$), 11.28 (br, 1H, salicyl-2-O*H*).

${}^{13}C\{{}^1H\}$NMR (100 MHz, $CDCl_3$, 23 °C): δ = 22.58 (NCH_2CH_2CH(*C*$H_3)_2$), 26.11 (NCH_2CH_2-*C*$H(CH_3)_2$), 38.17 (N*C*$H_2CH_2CH(CH_3)_2$), 38.51 (NCH_2*C*$H_2CH(CH_3)_2$), 49.08 (*C*-3,4 piperazine), 52.36 (*C*-2,6 piperazine), 60.46 (salicyl-3-*C*H_2-1-piperazine-4-phenyl), 116.44 (*C*-2,6 phenyl of 4-phenyl-piperazine), 120.54 (*C*-4 phenyl of 4-phenylpiperazine), 121.14 (C_q-1 salicyl), 123.30 (C_q-3 salicyl), 124.28 (C_q-5 salicyl), 129.29 (*C*-3,5 phenyl of 4-phenylpiperazine), 130.24 (*C*-6 salicyl), 131.39

(*C*-4 salicyl), 150.71 (C_q-1 phenyl of 4-phenylpiperazine), 155.59 (C_q-2 salicyl), 164.63 (*C*ONHCH$_2$CH$_2$-CH(CH$_3$)$_2$).

(12) 5-Chloro-3-(1,3-dioxo-1,3-dihydro-isoindol-2-ylmethyl)-2-hydroxy-*N*-(3-methyl-butyl)-benzamide

Colorless crystals, yield: 69%, mp: 174–176 °C.

^{1}H-NMR (400 MHz, CDCl$_3$, 23 °C): δ = 0.94 (d, ^3J = 6.5, 6H, CONHCH$_2$CH$_2$CH(C*H*$_3$)$_2$), 1.49–1.55 (m, 2H, CONHCH$_2$C*H*$_2$CH(CH$_3$)$_2$), 1.62–1.73 (m[sept], 1H, CONHCH$_2$CH$_2$C*H*(CH$_3$)$_2$), 3.41–3.48 (m, 2H, CONHC*H*$_2$CH$_2$CH(CH$_3$)$_2$), 4.89 (s, 2H, salicyl-3-C*H*$_2$-N-phthalimide), 6.57 (t, ^3J = 5.3, 1H, CON*H*CH$_2$CH$_2$CH(CH$_3$)$_2$), 7.16 (d, ^4J = 2.8, 1H, *H*-4 salicyl), 7.20 (d, ^4J = 2.8, 1H, *H*-6 salicyl), 7.71–7.77 (m, 2H, *H*-5,6, AA'-phthalimide), 7.84–7.90 (m, 2H, *H*-4,7 BB'-phthalimide), 12.72 (s, sharp, 1H, salicyl-2-O*H*).

^{13}C{^{1}H}NMR (100 MHz, CDCl$_3$, 23 °C): δ = 22.55 (NCH$_2$CH$_2$CH(*C*H$_3$)$_2$), 26.03 (NCH$_2$CH$_2$-*C*H(CH$_3$)$_2$), 36.24 (salicyl-3-*C*H$_2$-N-phthalimide), 38.24 (NCH$_2$*C*H$_2$CH(CH$_3$)$_2$), 38.41 (N*C*H$_2$CH$_2$-CH(CH$_3$)$_2$), 115.52 (C_q-1 salicyl), 123.02 (C_q-5 salicyl), 123.66 (*C*-4,7 phthalimide), 124.82 (*C*-6 salicyl), 126.84 (C_q-3 salicyl), 132.14 (*C*-3a,7a phthalimide), 132.35 (*C*-4 salicyl), 134.34 (*C*-5,6 phthalimide), 157.76 (C_q-2 salicyl), 168.29 (*C*O phthalimide), 168.74 (*C*ONHCH$_2$CH$_2$CH(CH$_3$)$_2$).

(13) 1-[5-Chloro-2-hydroxy-3-(3-methyl-butylcarbamoyl)-benzyl]-4-phenyl-piperidinium chloride

Colorless crystals, mp: 234–237 °C.

^{1}H-NMR (400 MHz, DMSO-d$_6$, 23 °C): δ = 0.91 (d, ^3J = 6.6, 6H, CONHCH$_2$CH$_2$CH(C*H*$_3$)$_2$), 1.42–1.49 (m, 2H, CONHCH$_2$C*H*$_2$CH(CH$_3$)$_2$), 1.56–1.67 (m[sept], 1H, CONHCH$_2$CH$_2$C*H*(CH$_3$)$_2$), 1.88–1.97 (m[d], 2H, CH_{eq}-3,5 piperidinium), 2.03–2.16 (m[62], 2H, CH_{ax}-3,5 piperidinium), 2.76–2.87 (m[t], 1H, CH_{ax}-4 piperidinium), 3.05–3.17 (m[t], 2H, CH_{ax}-2,6 piperidinium), 3.28–3.33 (overlay with water, m, 2H, CONHC*H*$_2$CH$_2$CH(CH$_3$)$_2$), 3.41–3.50 (m[d], 2H, CH_{eq}-2,6 piperidinium), 4.27 (s, br, 2H, salicyl-3-C*H*$_2$N$^+$H-piperidinium), 7.18–7.25 (m, 3H, *H*-2,6,4 phenyl in 4 phenylpiperidinium), 7.28–7.36 (m, 2H, *H*-3,5 phenyl in 4-phenylpiperidinium), 7.97 (d, ^4J = 2.4, 1H, *H*-4 salicyl), 8.21 (d, ^4J = 2.5, 1H, *H*-6 salicyl), 9.29 (t, ^3J = 5.3, 1H, CON*H*CH$_2$CH$_2$CH(CH$_3$)$_2$), 10.84 (s, br, 1H, 3-salicyl-CH$_2$N$^+$*H*-piperidinium), 14.10 (br, 1H, salicyl-2-O*H*).

^{13}C{^{1}H}NMR (100 MHz, DMSO-d$_6$, 23 °C): δ = 22.31 (CONHCH$_2$CH$_2$CH(*C*H$_3$)$_2$), 25.21 (CONH-CH$_2$CH$_2$*C*H(CH$_3$)$_2$), 29.65 (*C*-3,5 piperidinium), 37.42 (CONHCH$_2$*C*H$_2$CH(CH$_3$)$_2$), 37.48 (CONH-*C*H$_2$CH$_2$CH(CH$_3$)$_2$), 38.49 (*C*-4 piperidinium), 51.71 (*C*-2,6 piperidinium), 52.10 (salicyl-3-*C*H$_2$-4-phenylpiperidinium), 115.56 (C_q-3 salicyl), 120.03 (C_q-1 salicyl), 121.75 (C_q-5 salicyl), 126.52 (*C*-2,6 phenyl in 4-phenylpiperidinium, *C*-4 phenyl in 4-phenylpiperidinium), 128.21 (*C*-6 salicyl), 128.52 (*C*-3,5 phenyl in 4-phenylpiperidinium), 137.15 (*C*-4 salicyl), 144.31 (C_q-1 phenyl in 4-phenyl-piperidinium), 159.33 (C_q-2 salicyl), 168.47 (*C*ONHCH$_2$CH$_2$CH(CH$_3$)$_2$).

(**14**) Diethyl-[2-hydroxy-3-(3-methyl-butylcarbamoyl)-benzyl]-ammonium chloride

Colorless crystals, mp: 172–174 °C.

^{1}H-NMR (400 MHz, DMSO-d_6, 23 °C): δ = 0.88 (d, 6H, $CONHCH_2CH_2CH(C\mathit{H}_3)_2$), 1.24 (t, 6H, $NH^+(CH_2C\mathit{H}_3)_2$), 1.40–1.46 (m, 2H, $CONHCH_2C\mathit{H}_2CH(CH_3)_2$), 1.58 (sept, 1H, $CONHCH_2CH_2$-$C\mathit{H}(CH_3)_2$), 3.00–3.14 (m, 4H, $NH^+(C\mathit{H}_2CH_3)_2$), 3.28–3.33 (m, 2H, $CONHC\mathit{H}_2CH_2CH(CH_3)_2$), 4.23 (s, 2H, salicyl-3-$C\mathit{H}_2$-$C\mathit{H}_2NH^+(CH_2CH_3)_2$), 6.95 (dd[t], 2 × ^3J = 7.6, 1H, *H*-5 salicyl), 7.68 (dd, ^3J = 6.9, ^4J = 0.9, 1H, *H*-4 salicyl), 7.99 (dd, ^3J = 7.9, ^4J = 1.1, 1H, *H*-6 salicyl), 9.15 (t, ^3J = 5.5, 1H, $CON\mathit{H}CH_2CH_2CH(CH_3)_2$), 9.44 (s, br, 1H, $CH_2N^+\mathit{H}(CH_2CH_3)_2$), 14.00 (s, br, 1H, salicyl-2-O*H*).

^{13}C{^{1}H}NMR (100 MHz, DMSO-d_6, 23 °C): δ = 8.72 ($CH_2N^+H(CH_2\mathit{C}H_3)_2$), 22.62 ($NCH_2CH_2$-$CH(\mathit{C}H_3)_2$), 25.57 ($NCH_2CH_2\mathit{C}H(CH_3)_2$), 37.65 ($N\mathit{C}H_2CH_2CH(CH_3)_2$), 37.86 ($NCH_2\mathit{C}H_2CH(CH_3)_2$), 46.94 ($CH_2N^+H(\mathit{C}H_2CH_3)_2$), 49.66 ($\mathit{C}H_2N^+H(CH_2CH_3)_2$), 114.66 ($C_q$-3 salicyl), 118.64 (*C*-5 salicyl), 118.75 (C_q-1 salicyl), 128.99 (*C*-6 salicyl), 137.36 (*C*-4 salicyl), 160.44 (C_q-2 salicyl), 169.79 (*C*ONH).

(**15**) [5-Chloro-2-hydroxy-3-(3-methyl-butylcarbamoyl)-benzyl]-diethyl-ammonium chloride

Colorless crystals, mp: 224–227 °C.

^{1}H-NMR (400 MHz, DMSO-d_6, 23 °C): δ = 0.90 (d, ^3J = 6.6, 6H, $CONHCH_2CH_2CH(C\mathit{H}_3)_2$), 1.23–1.29 (m, 2H, $CONHCH_2C\mathit{H}_2CH(CH_3)_2$), 1.42–1.49 (t, 6H, $NH^+(CH_2C\mathit{H}_3)_2$), 1.56–1.67 (m[sept], 1H, $CONHCH_2CH_2C\mathit{H}(CH_3)_2$), 2.99–3.17 (m, 4H, $NH^+(C\mathit{H}_2CH_3)_2$), 3.28–3.35 (m, 2H, $CONHC\mathit{H}_2CH_2$-$CH(CH_3)_2$), 4.25 (d, ^3J = 4.5, 2H, salicyl-3-$C\mathit{H}_2$-$N^+H(CH_2CH_3)_2$), 7.96 (d, ^4J = 2.4, 1H, *H*-4 salicyl), 8.21 (d, ^4J = 2.8, 1H, *H*-6 salicyl), 9.32 (br[t], 1H, $CON\mathit{H}CH_2CH_2CH(CH_3)_2$), 10.30 (s, br, 1H, 3-salicyl-CH_2 $N^+\mathit{H}(CH_2CH_3)_2$), 14.13 (s, sharp, 1H, salicyl-2-O*H*).

^{13}C{^{1}H}NMR (100 MHz, DMSO-d_6, 23 °C): δ = 8.34 ($CH_2N^+H(CH_2\mathit{C}H_3)_2$), 22.30 ($NCH_2CH_2$-$CH(\mathit{C}H_3)_2$), 25.20 ($NCH_2CH_2\mathit{C}H(CH_3)_2$), 37.40 ($NCH_2\mathit{C}H_2CH(CH_3)_2$), 37.47 ($N\mathit{C}H_2CH_2CH(CH_3)_2$), 46.21 (salicyl-3-$CH_2N^+H(\mathit{C}H_2CH_3)_2$), 48.21 (salicyl-3-$\mathit{C}H_2N^+H(CH_2CH_3)_2$), 115.48 ($C_q$-3 salicyl), 120.64 ($C_q$-1 salicyl), 121.82 ($C_q$-5 salicyl), 128.10 (*C*-6 salicyl), 136.63 (*C*-4 salicyl), 159.10 (C_q-2 salicyl), 168.44 ($\mathit{C}ONHCH_2CH_2CH(CH_3)_2$).

4. Representation of the $^{1}H/^{13}C$ NMR Spectra of Compounds 1–15

(1) 2-Hydroxy-*N*-(3-methyl-butyl)-benzamide

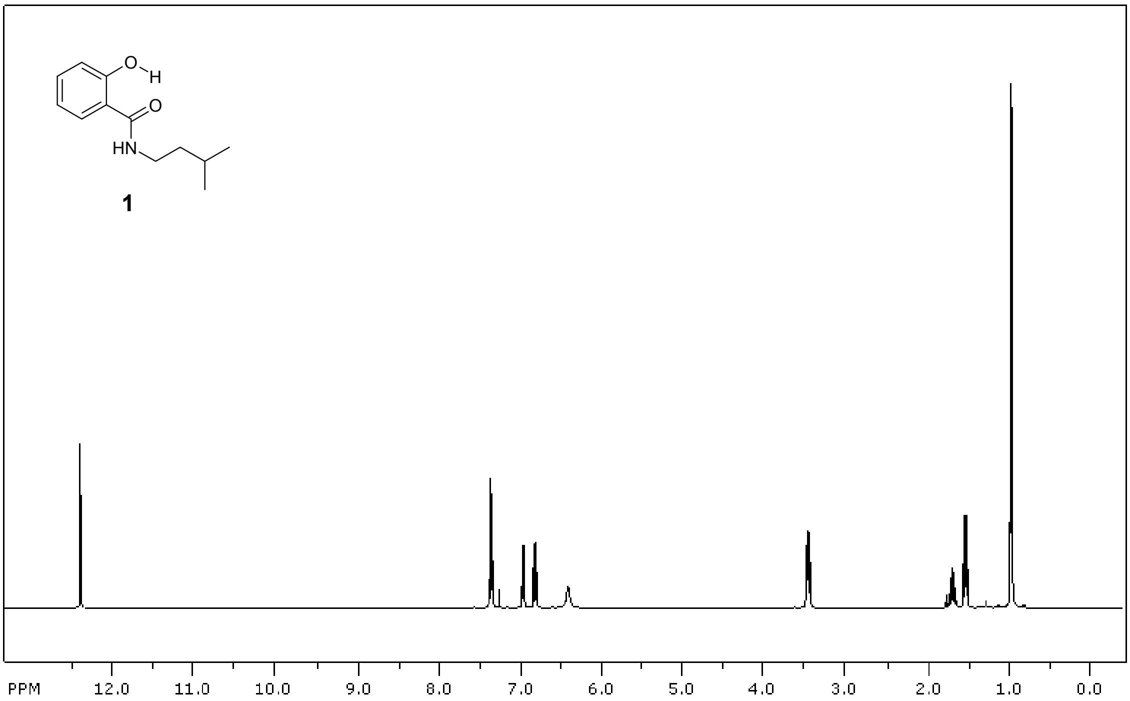

file: ...57,MM195,MM247,MM244\130_MM195\fid expt: <zg30>
transmitter freq.: 400.132471 MHz
time domain size: 65536 points
width: 8278.15 Hz = 20.6885 ppm = 0.126314 Hz/pt
number of scans: 16

freq. of 0 ppm: 400.130009 MHz
processed size: 32768 complex points
LB: 0.300 GF: 0.0000
Hz/cm: 220.629 ppm/cm: 0.55139

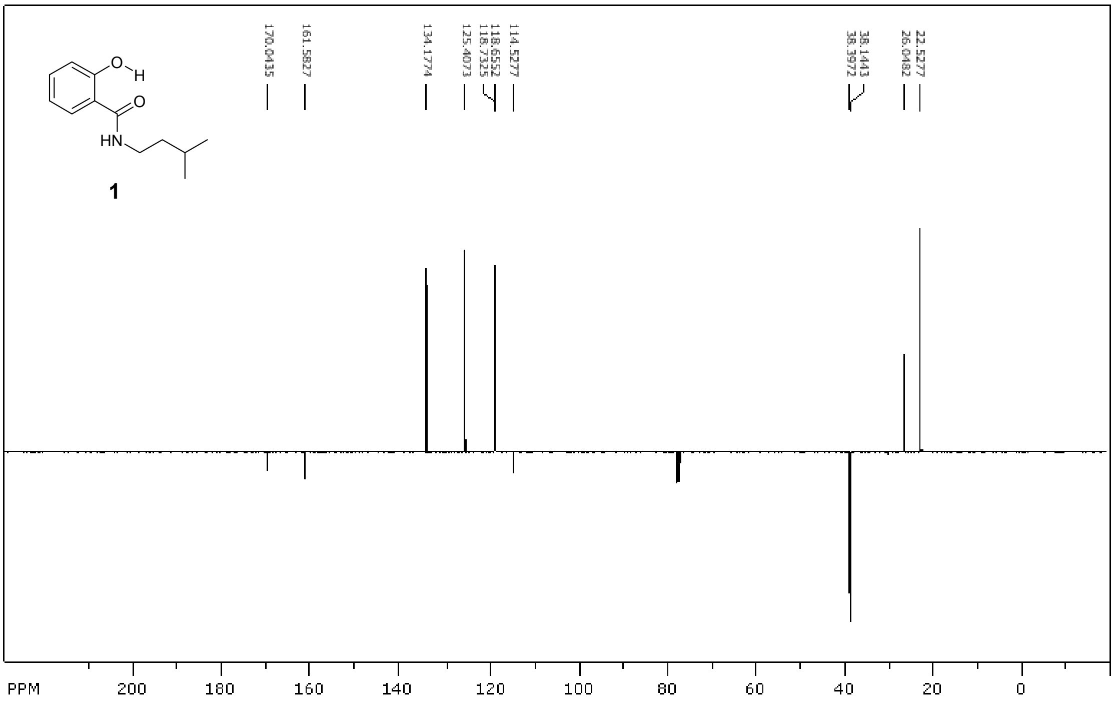

file: ...53,MM157,MM195,MM247,MM244\131\fid expt: <jmod>
transmitter freq.: 100.623333 MHz
time domain size: 65536 points
width: 25062.66 Hz = 249.0740 ppm = 0.382426 Hz/pt
number of scans: 1000

freq. of 0 ppm: 100.612758 MHz
processed size: 32768 complex points
LB: 1.000 GF: 0.0000
Hz/cm: 1002.506 ppm/cm: 9.96296

(2) 5-Diethylaminomethyl-2-hydroxy-*N*-(3-methyl-butyl)-benzamide

SpinWorks 3: Auftraggeber MUW

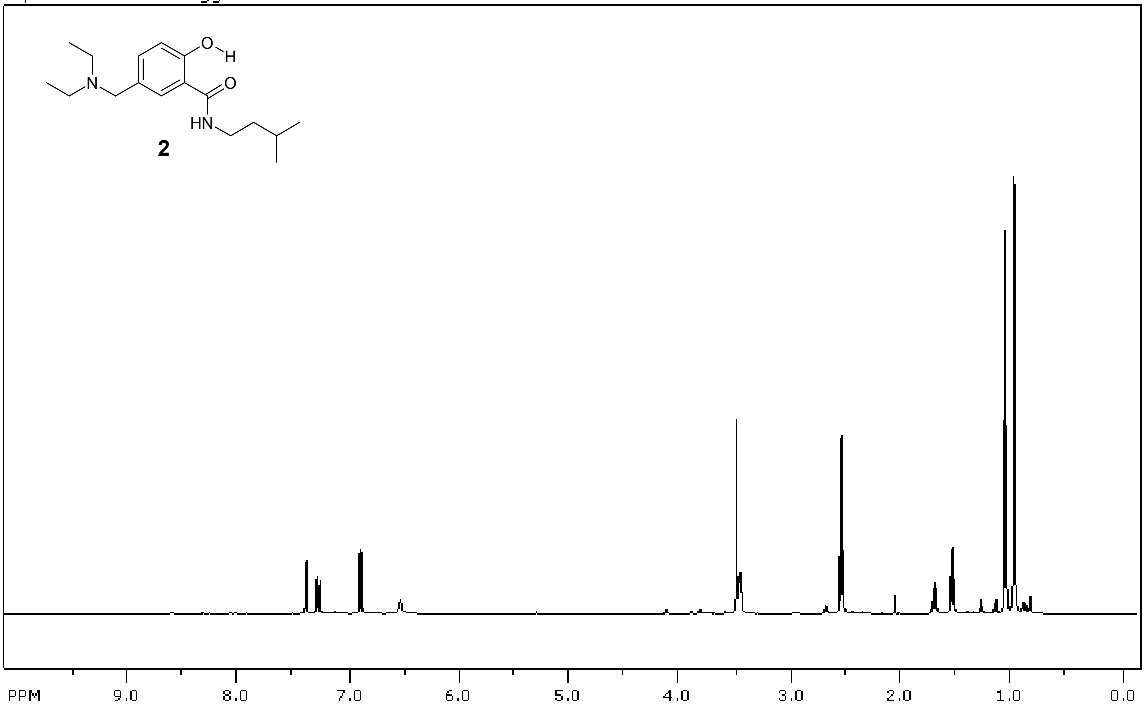

file: ...NMRrawdata\6Jan2214_HG2-7F2\30\fid expt: <zg30>
transmitter freq.: 600.133706 MHz
time domain size: 65536 points
width: 12335.53 Hz = 20.5546 ppm = 0.188225 Hz/pt
number of scans: 16

freq. of 0 ppm: 600.130017 MHz
processed size: 65536 complex points
LB: 0.300 GF: 0.0000
Hz/cm: 246.981 ppm/cm: 0.41154

SpinWorks 3: Auftraggeber MUW

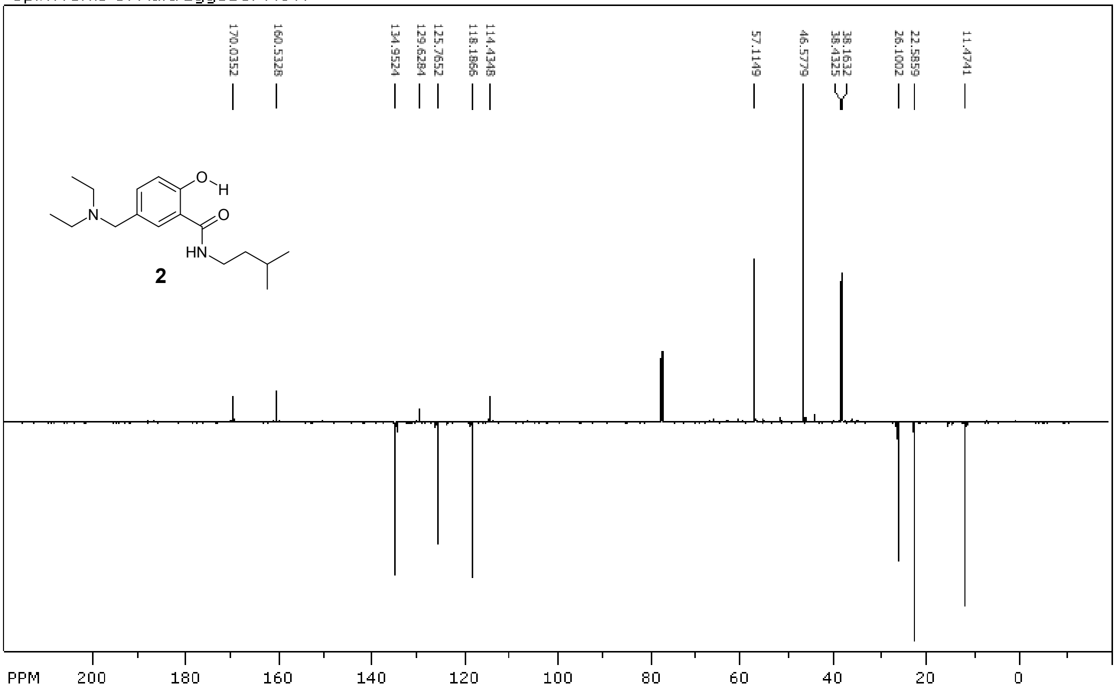

file: ...NMRrawdata\6Jan2214_HG2-7F2\34\fid expt: <deptqgpsp.2>
transmitter freq.: 150.917899 MHz
time domain size: 65536 points
width: 36057.69 Hz = 238.9226 ppm = 0.550197 Hz/pt
number of scans: 1024

freq. of 0 ppm: 150.902790 MHz
processed size: 131072 complex points
LB: 1.000 GF: 0.0000
Hz/cm: 1442.308 ppm/cm: 9.55690

(3) 3-Diethylaminomethyl-2-hydroxy-*N*-(3-methyl-butyl)-benzamide

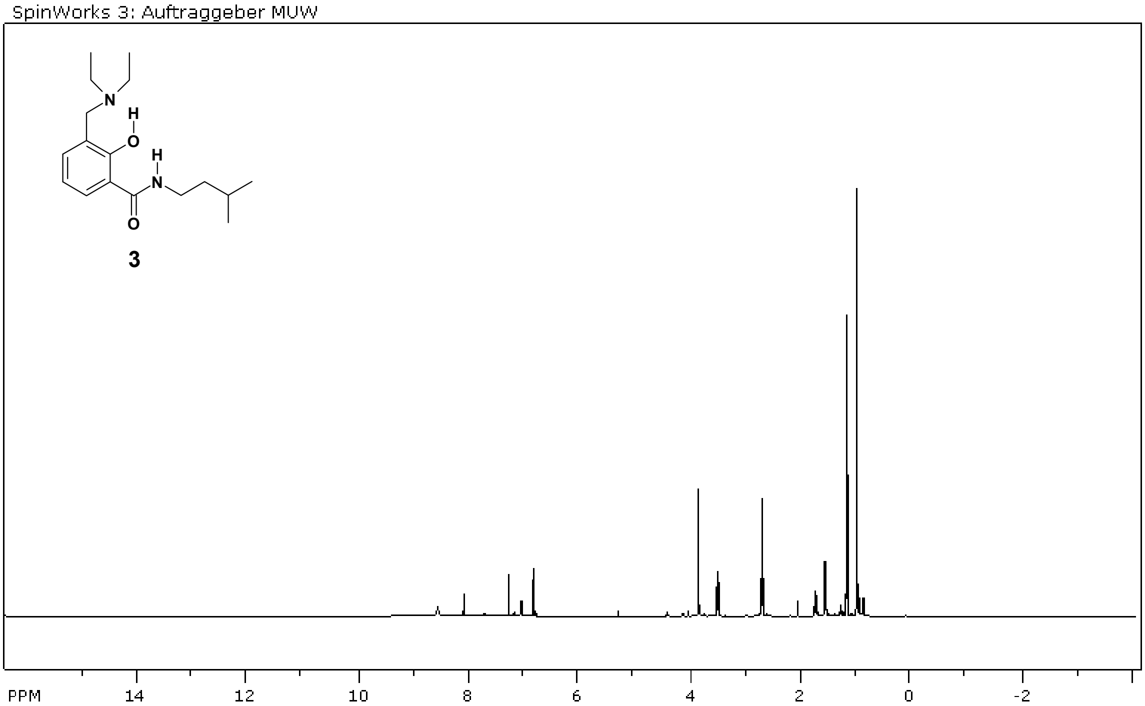

file: ...NMRrawdata\6Jan2214_HG2-7F1\20\fid expt: <zg30>
transmitter freq.: 600.133706 MHz
time domain size: 65536 points
width: 12335.53 Hz = 20.5546 ppm = 0.188225 Hz/pt
number of scans: 16

freq. of 0 ppm: 600.130018 MHz
processed size: 65536 complex points
LB: 0.300 GF: 0.0000
Hz/cm: 493.421 ppm/cm: 0.82219

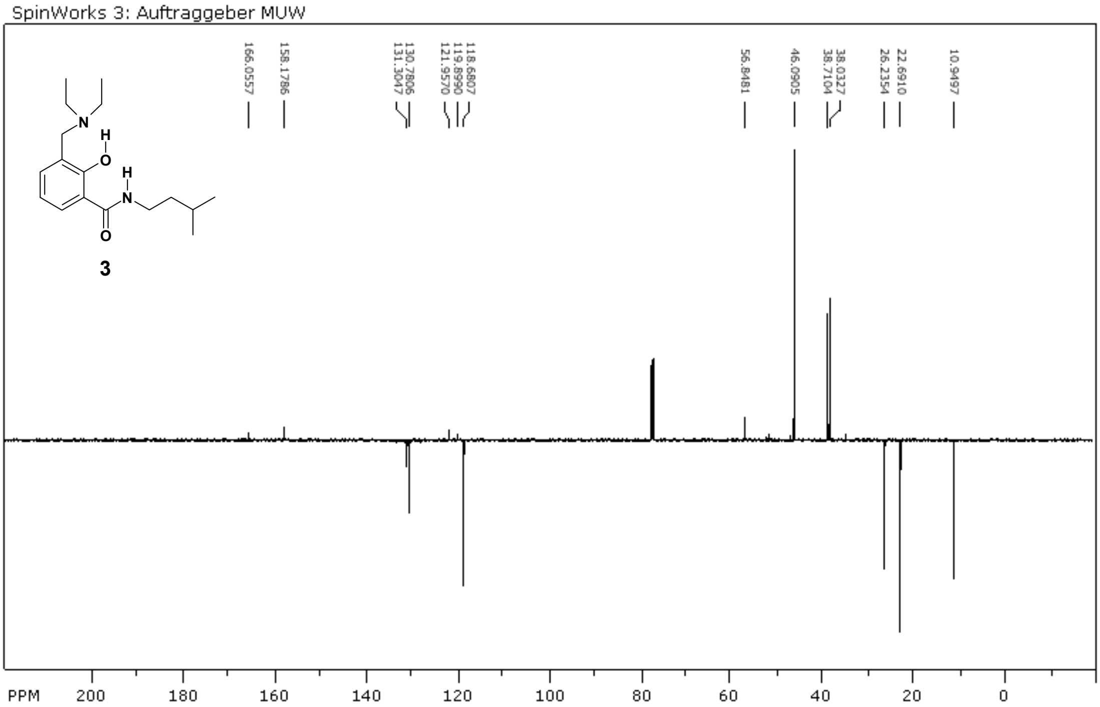

file: ...NMRrawdata\6Jan2214_HG2-7F1\24\fid expt: <deptqgpsp.2>
transmitter freq.: 150.917899 MHz
time domain size: 65536 points
width: 36057.69 Hz = 238.9226 ppm = 0.550197 Hz/pt
number of scans: 532

freq. of 0 ppm: 150.902790 MHz
processed size: 131072 complex points
LB: 1.000 GF: 0.0000
Hz/cm: 1442.308 ppm/cm: 9.55690

(4) 3,5-Bis-diethylaminomethyl-2-hydroxy-*N*-(3-methyl-butyl)-benzamide

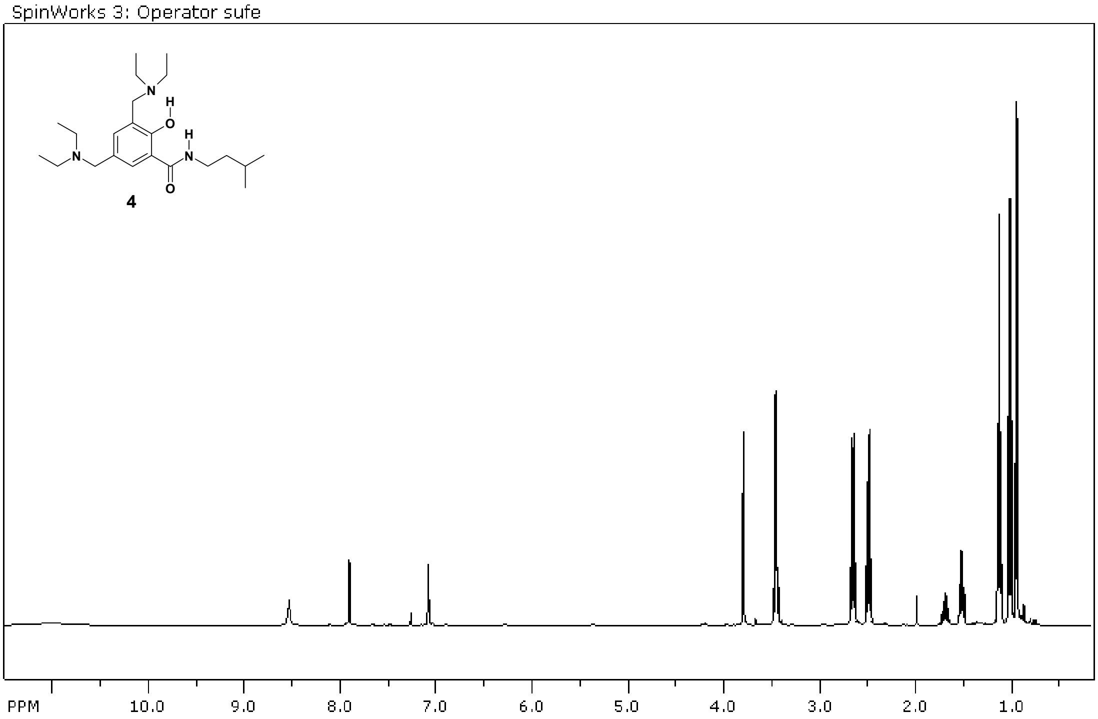

file: ...b2414_GM177_BIG-1 purified\290\fid expt: <zg30>
transmitter freq.: 400.272472 MHz
time domain size: 65536 points
width: 8012.82 Hz = 20.0184 ppm = 0.122266 Hz/pt
number of scans: 16

freq. of 0 ppm: 400.270011 MHz
processed size: 65536 complex points
LB: 0.300 GF: 0.0000
Hz/cm: 182.245 ppm/cm: 0.45530

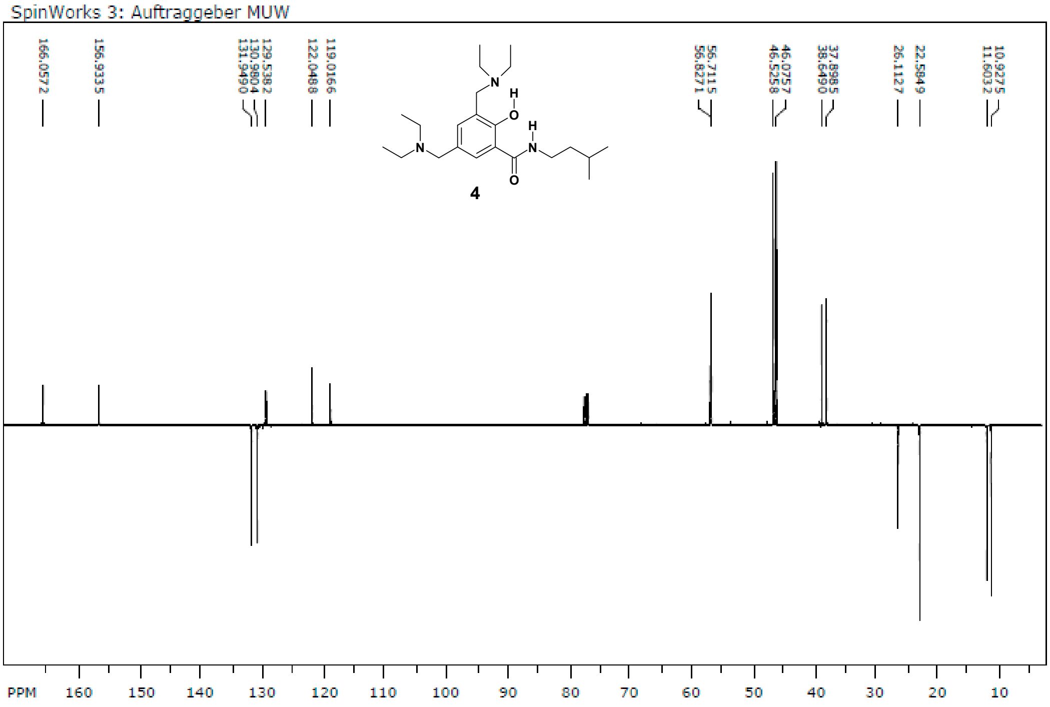

file: ...rawdata\Feb2414_BK012_GM177\14\fid expt: <jmod>
transmitter freq.: 100.623333 MHz
time domain size: 65536 points
width: 25062.66 Hz = 249.0740 ppm = 0.382426 Hz/pt
number of scans: 800

freq. of 0 ppm: 100.612762 MHz
processed size: 32768 complex points
LB: 1.000 GF: 0.0000
Hz/cm: 683.377 ppm/cm: 6.79144

(5) 5-Chloro-2-hydroxy-*N*-(3-methyl-butyl)-benzamide

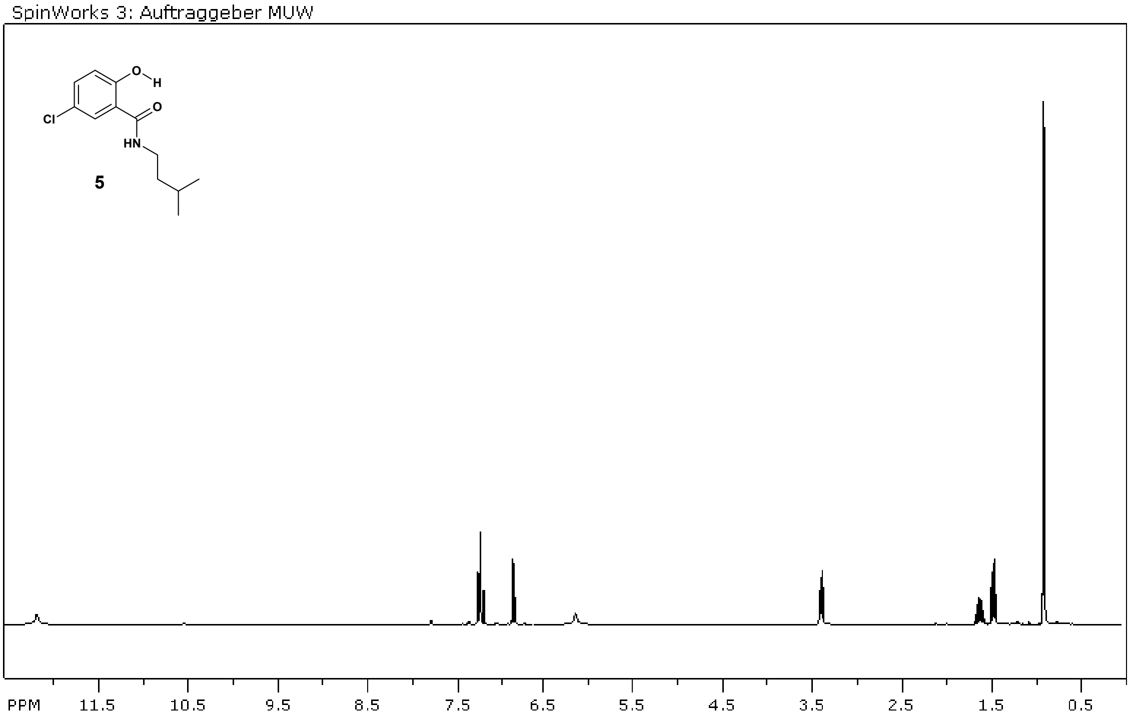

file: ...MR\NMRrawdata\Feb0714_BK001\10\fid expt: <zg30>
transmitter freq.: 400.132471 MHz
time domain size: 65536 points
width: 8278.15 Hz = 20.6885 ppm = 0.126314 Hz/pt
number of scans: 16

freq. of 0 ppm: 400.130037 MHz
processed size: 32768 complex points
LB: 0.300 GF: 0.0000
Hz/cm: 201.365 ppm/cm: 0.50325

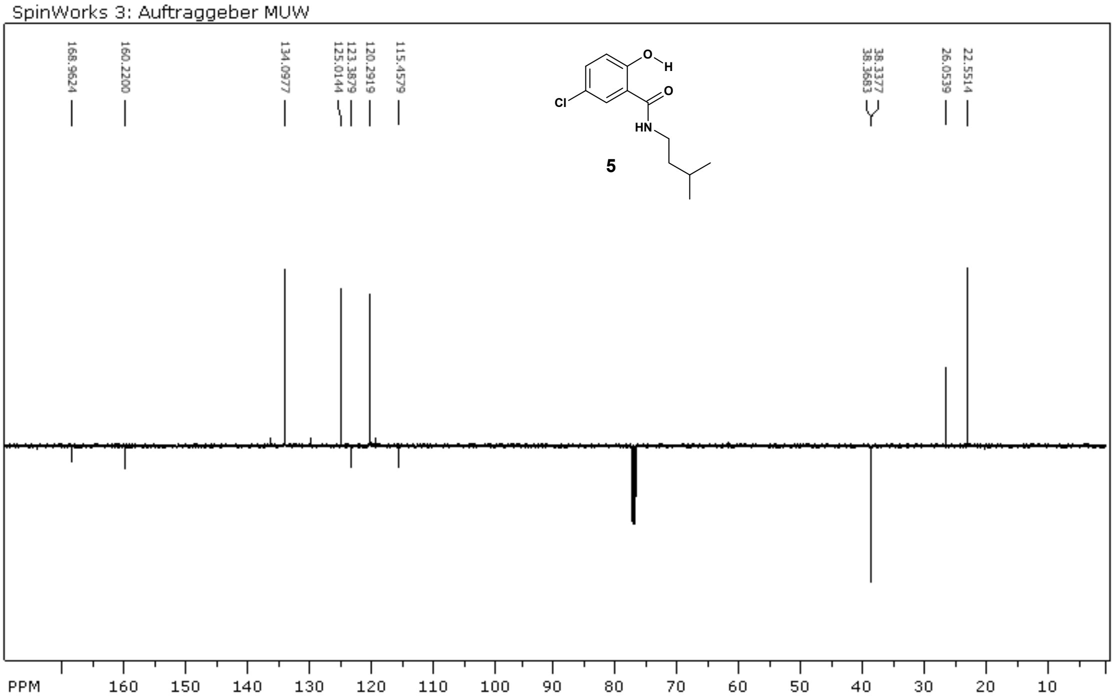

file: ...MR\NMRrawdata\Feb0714_BK001\11\fid expt: <jmod>
transmitter freq.: 100.623333 MHz
time domain size: 65536 points
width: 25062.66 Hz = 249.0740 ppm = 0.382426 Hz/pt
number of scans: 800

freq. of 0 ppm: 100.612754 MHz
processed size: 32768 complex points
LB: 1.000 GF: 0.0000
Hz/cm: 724.093 ppm/cm: 7.19608

(6) 5-Chloro-3-diethylaminomethyl-2-hydroxy-*N*-(3-methyl-butyl)-benzamide

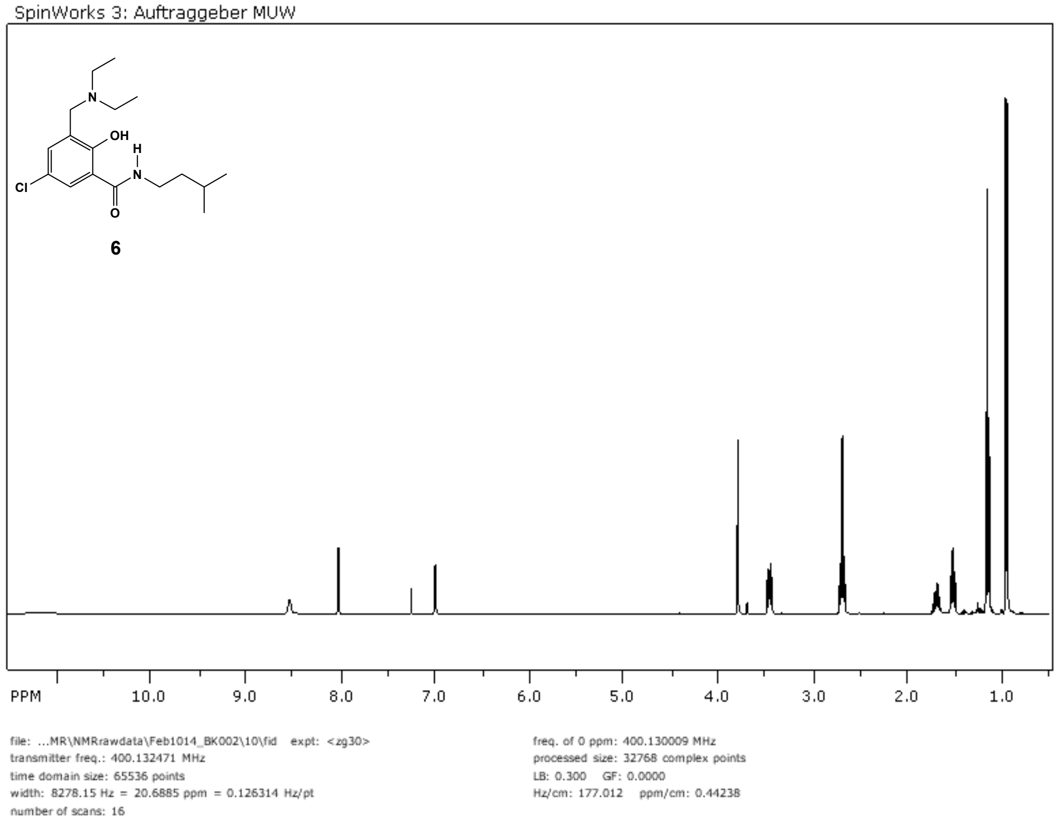

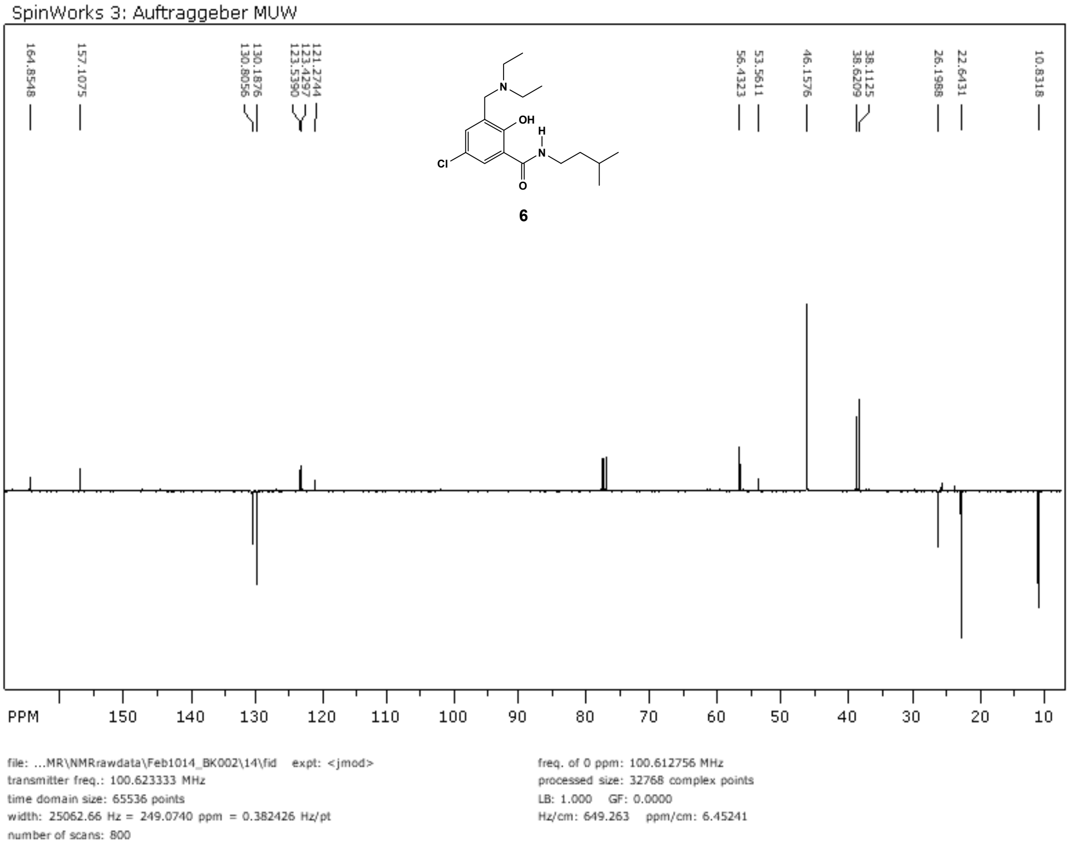

(7) 5-Chloro-3-dibutylaminomethyl-2-hydroxy-*N*-(3-methyl-butyl)-benzamide

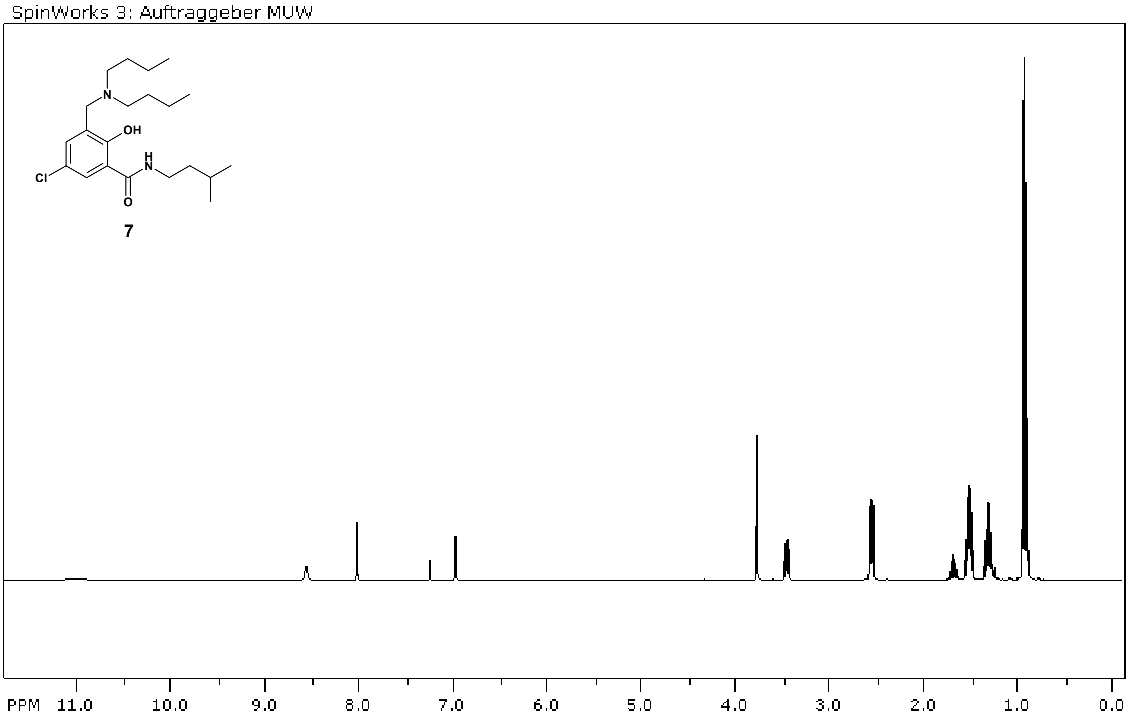

file: ...MR\NMRrawdata\Feb2014_BK011\60\fid expt: <zg30>
transmitter freq.: 400.132471 MHz
time domain size: 65536 points
width: 8278.15 Hz = 20.6885 ppm = 0.126314 Hz/pt
number of scans: 16

freq. of 0 ppm: 400.130009 MHz
processed size: 32768 complex points
LB: 0.300 GF: 0.0000
Hz/cm: 190.461 ppm/cm: 0.47599

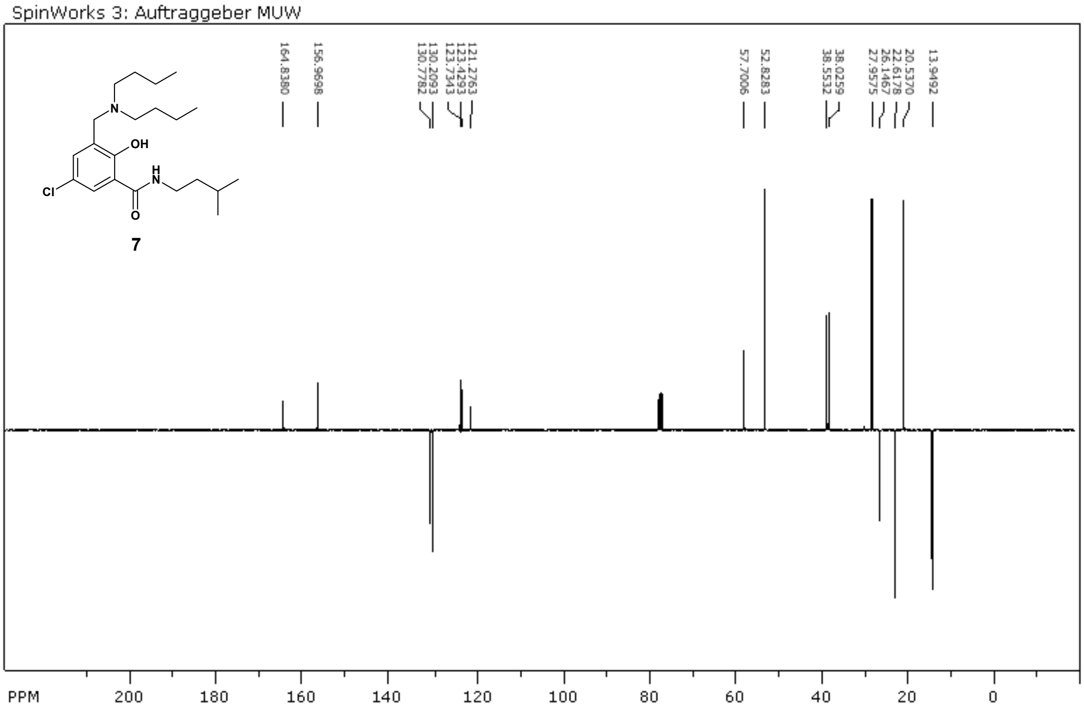

file: ...MR\NMRrawdata\Feb2014_BK011\64\fid expt: <jmod>
transmitter freq.: 100.623333 MHz
time domain size: 65536 points
width: 25062.66 Hz = 249.0740 ppm = 0.382426 Hz/pt
number of scans: 2500

freq. of 0 ppm: 100.612756 MHz
processed size: 32768 complex points
LB: 1.000 GF: 0.0000
Hz/cm: 1002.506 ppm/cm: 9.96296

(8) 5-Chloro-2-hydroxy-*N*-(3-methyl-butyl)-3-piperidin-1-ylmethyl-benzamide

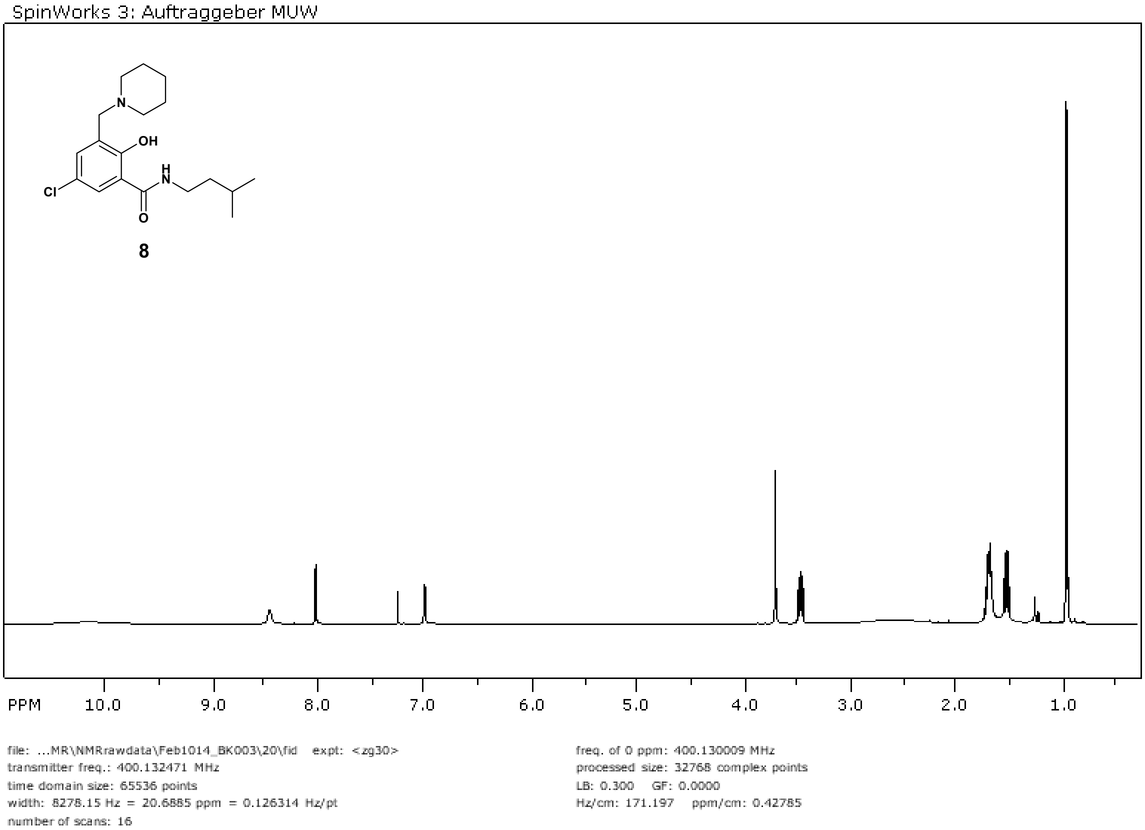

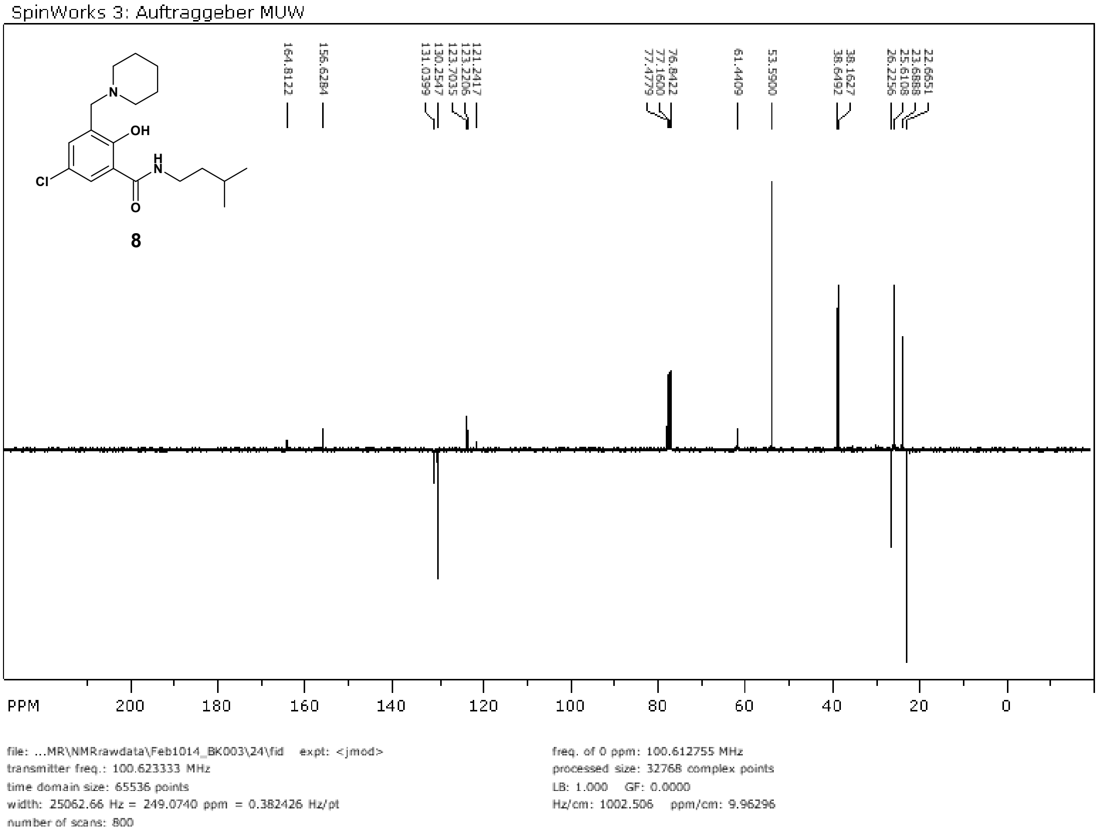

(9) 5-Chloro-2-hydroxy-*N*-(3-methyl-butyl)-3-(4-phenyl-piperidin-1-ylmethyl)-benzamide

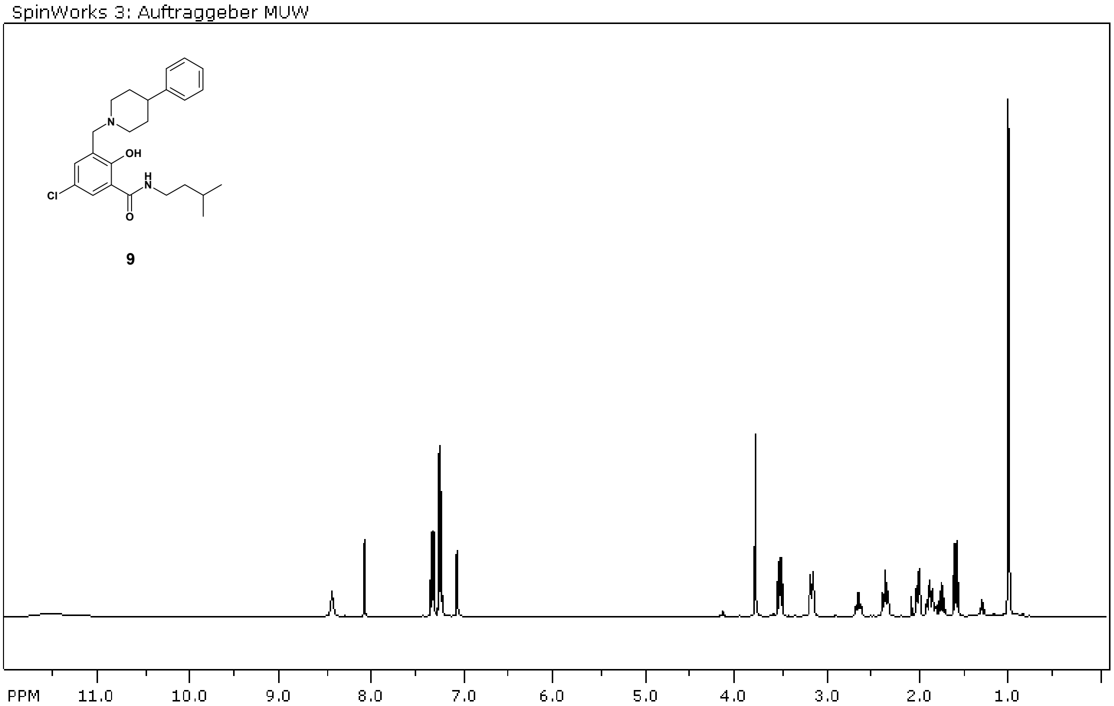

file: ...MR\NMRrawdata\Feb2014_BK010\50\fid expt: <zg30>
transmitter freq.: 400.132471 MHz
time domain size: 65536 points
width: 8278.15 Hz = 20.6885 ppm = 0.126314 Hz/pt
number of scans: 16

freq. of 0 ppm: 400.130003 MHz
processed size: 32768 complex points
LB: 0.300 GF: 0.0000
Hz/cm: 194.459 ppm/cm: 0.48599

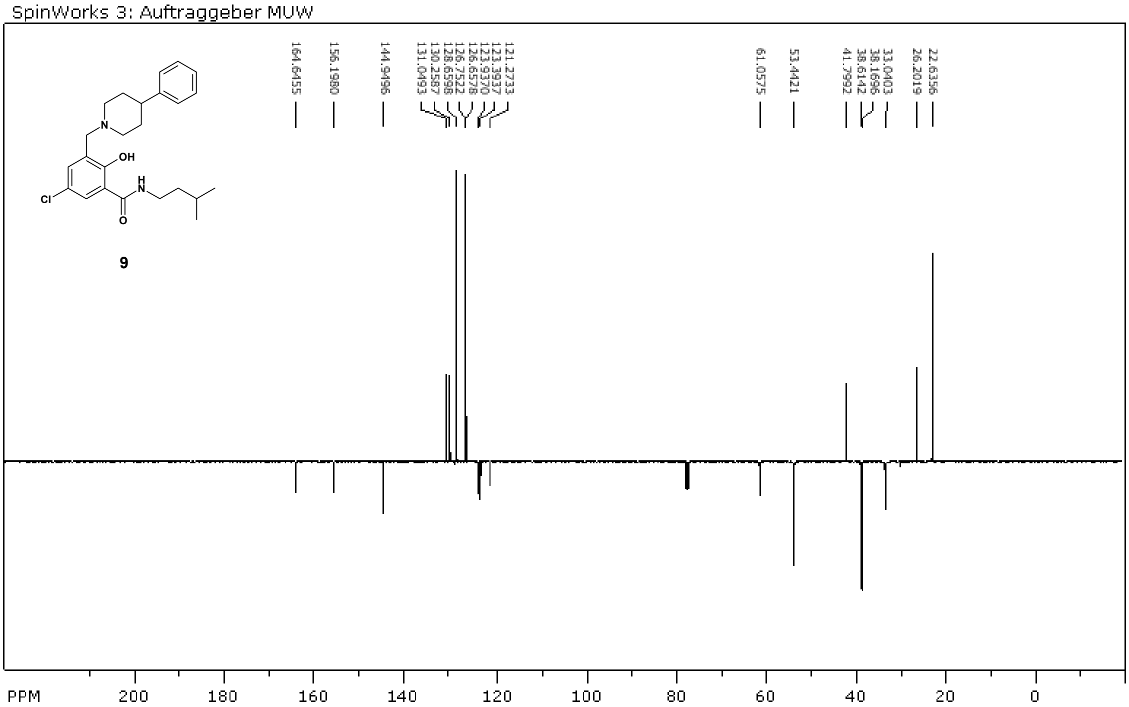

file: ...MR\NMRrawdata\Feb2014_BK010\54\fid expt: <jmod>
transmitter freq.: 100.623333 MHz
time domain size: 65536 points
width: 25062.66 Hz = 249.0740 ppm = 0.382426 Hz/pt
number of scans: 2500

freq. of 0 ppm: 100.612762 MHz
processed size: 32768 complex points
LB: 1.000 GF: 0.0000
Hz/cm: 1002.506 ppm/cm: 9.96296

(10) 3-(4-Benzyl-piperidin-1-ylmethyl)-5-chloro-2-hydroxy-*N*-(3-methyl-butyl)-benzamide

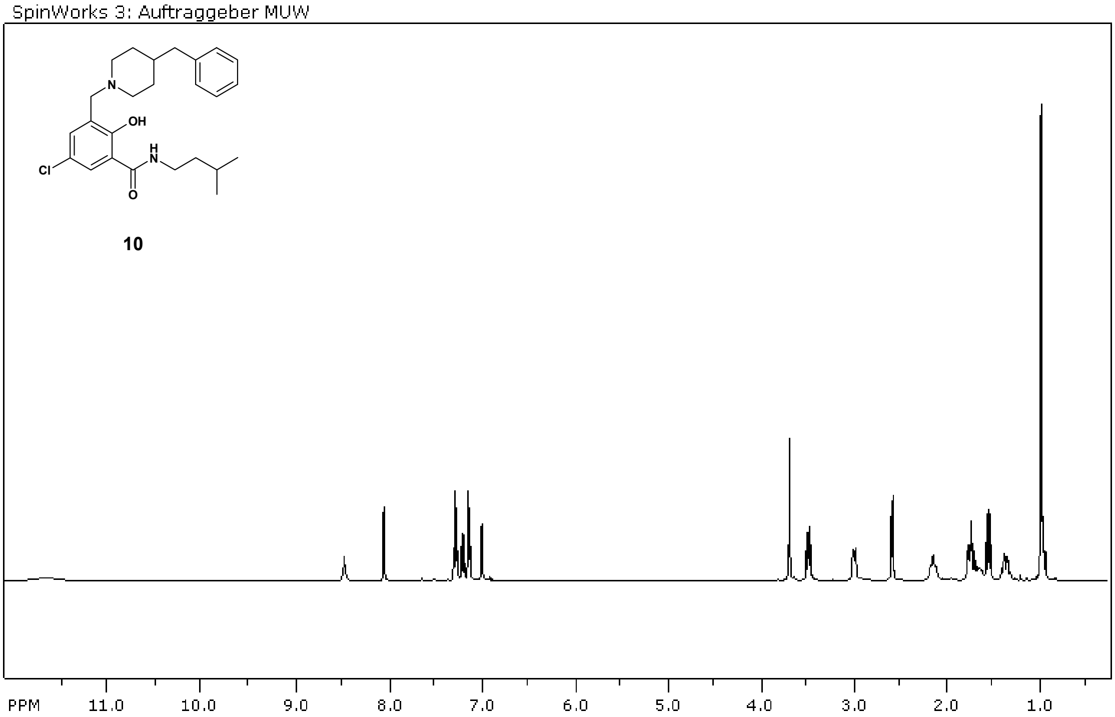

file: ...MR\NMRrawdata\Apr0214_BK026\10\fid expt: <zg30>
transmitter freq.: 400.132471 MHz
time domain size: 65536 points
width: 8278.15 Hz = 20.6885 ppm = 0.126314 Hz/pt
number of scans: 16

freq. of 0 ppm: 400.130009 MHz
processed size: 32768 complex points
LB: 0.300 GF: 0.0000
Hz/cm: 190.097 ppm/cm: 0.47509

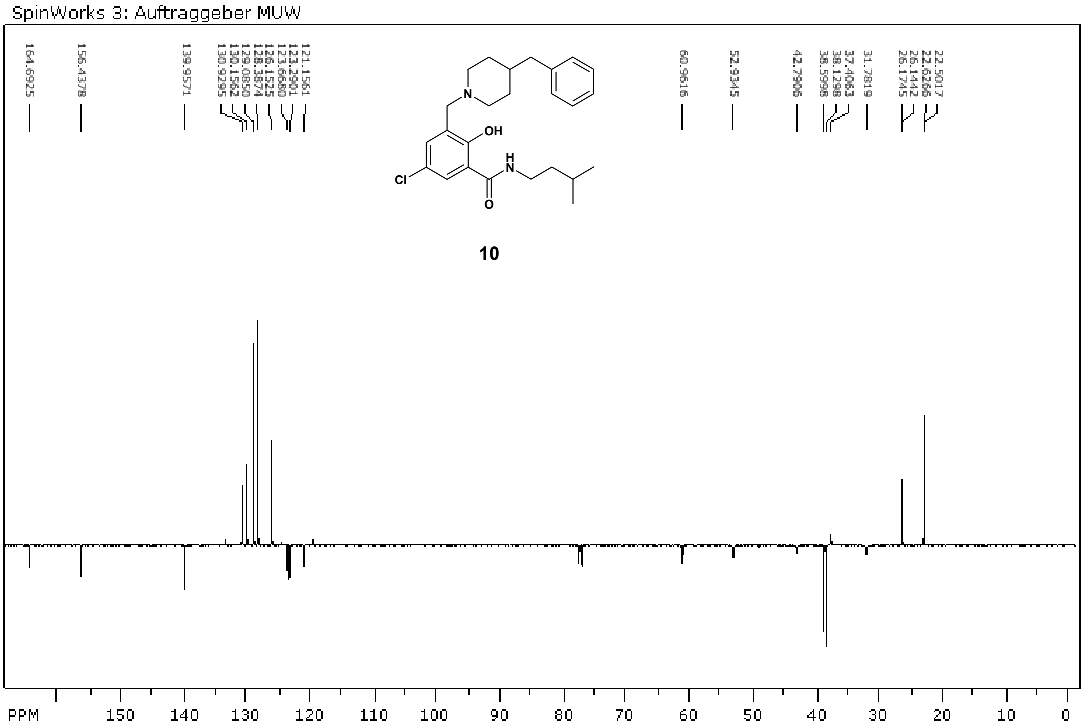

file: ...MR\NMRrawdata\Apr0214_BK026\14\fid expt: <jmod>
transmitter freq.: 100.623333 MHz
time domain size: 65536 points
width: 25062.66 Hz = 249.0740 ppm = 0.382426 Hz/pt
number of scans: 1200

freq. of 0 ppm: 100.612764 MHz
processed size: 32768 complex points
LB: 1.000 GF: 0.0000
Hz/cm: 684.477 ppm/cm: 6.80237

(11) 5-Chloro-2-hydroxy-*N*-(3-methyl-butyl)-3-(4-phenyl-piperazin-1-ylmethyl)-benzamide

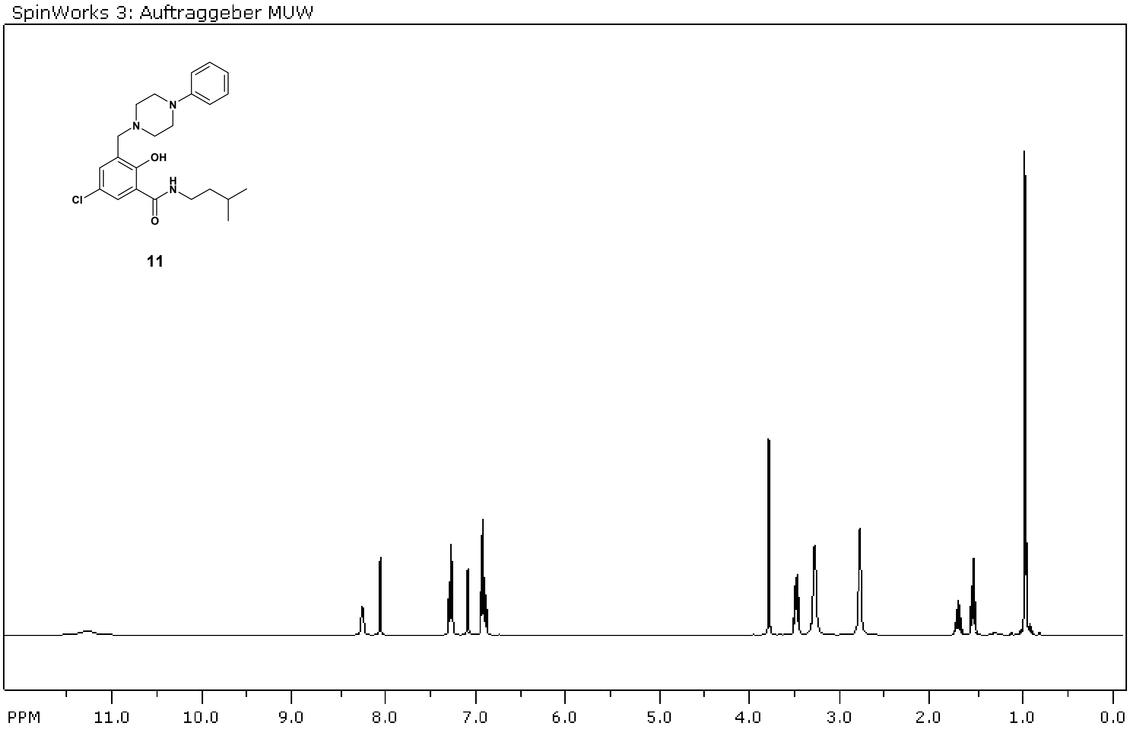

file: ...MR\NMRrawdata\Mar1214_BK017\41\fid expt: <zg30>
transmitter freq.: 400.132471 MHz
time domain size: 65536 points
width: 8278.15 Hz = 20.6885 ppm = 0.126314 Hz/pt
number of scans: 16

freq. of 0 ppm: 400.130009 MHz
processed size: 32768 complex points
LB: 0.300 GF: 0.0000
Hz/cm: 197.367 ppm/cm: 0.49325

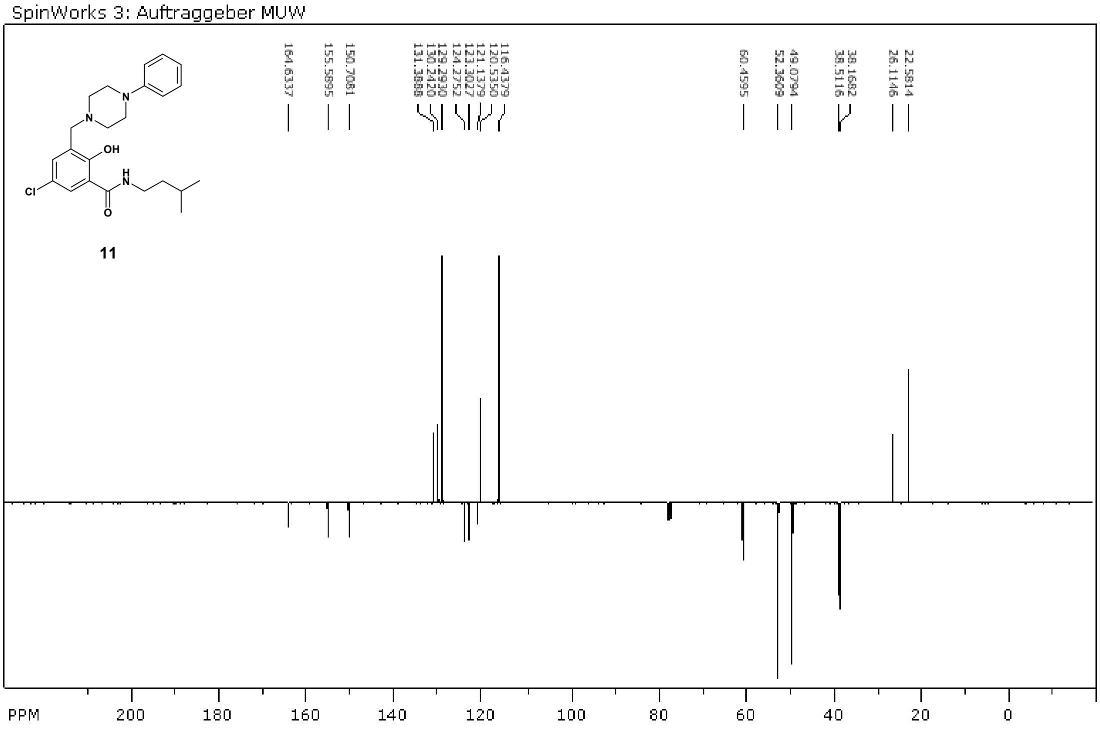

file: ...MR\NMRrawdata\Mar1214_BK017\40\fid expt: <jmod>
transmitter freq.: 100.623333 MHz
time domain size: 65536 points
width: 25062.66 Hz = 249.0740 ppm = 0.382426 Hz/pt
number of scans: 1000

freq. of 0 ppm: 100.612764 MHz
processed size: 32768 complex points
LB: 1.000 GF: 0.0000
Hz/cm: 1002.506 ppm/cm: 9.96296

(12) 5-Chloro-3-(1,3-dioxo-1,3-dihydro-isoindol-2-ylmethyl)-2-hydroxy-*N*-(3-methyl-butyl)-benzamide

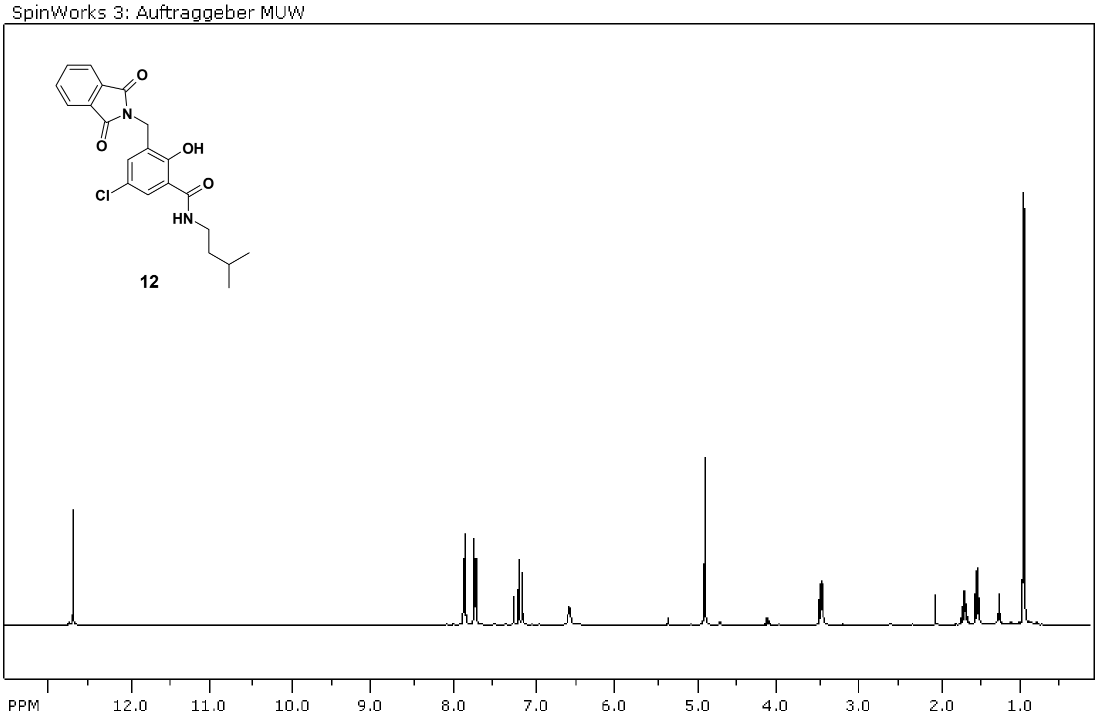

file: ...MR\NMRrawdata\Feb1114_BK004\10\fid expt: <zg30>
transmitter freq.: 400.132471 MHz
time domain size: 65536 points
width: 8278.15 Hz = 20.6885 ppm = 0.126314 Hz/pt
number of scans: 16

freq. of 0 ppm: 400.130009 MHz
processed size: 32768 complex points
LB: 0.300 GF: 0.0000
Hz/cm: 215.177 ppm/cm: 0.53777

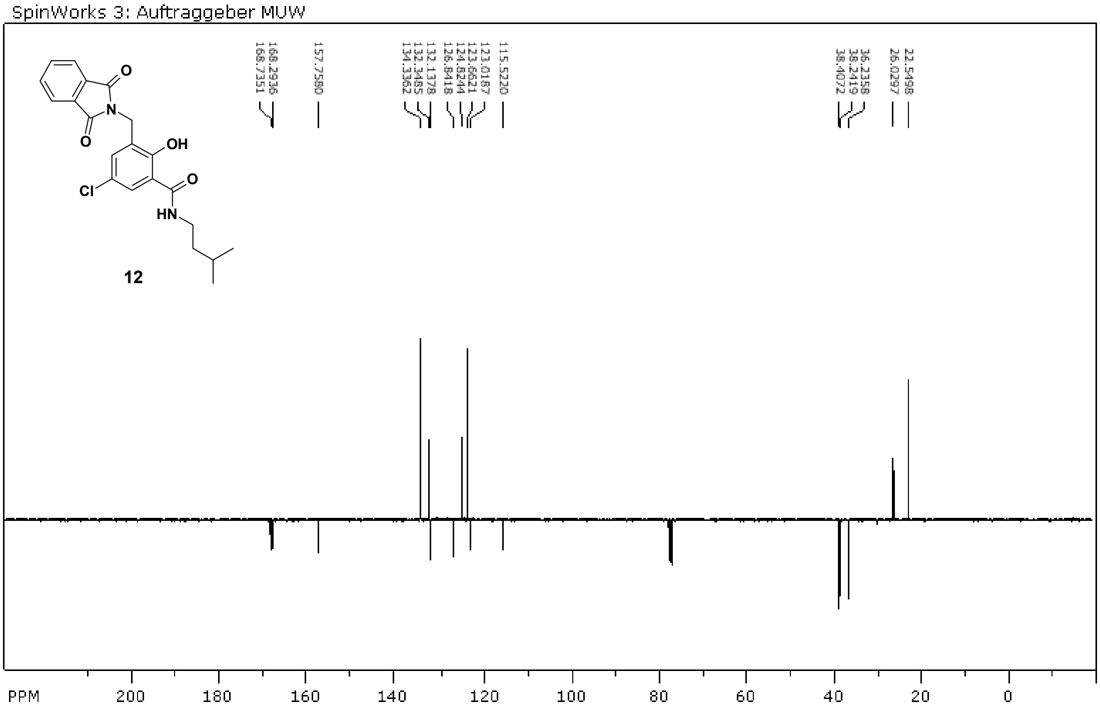

file: ...MR\NMRrawdata\Feb1114_BK004\14\fid expt: <jmod>
transmitter freq.: 100.623333 MHz
time domain size: 65536 points
width: 25062.66 Hz = 249.0740 ppm = 0.382426 Hz/pt
number of scans: 800

freq. of 0 ppm: 100.612755 MHz
processed size: 32768 complex points
LB: 1.000 GF: 0.0000
Hz/cm: 1002.506 ppm/cm: 9.96296

(13) 1-[5-Chloro-2-hydroxy-3-(3-methyl-butylcarbamoyl)-benzyl]-4-phenyl-piperidinium chloride

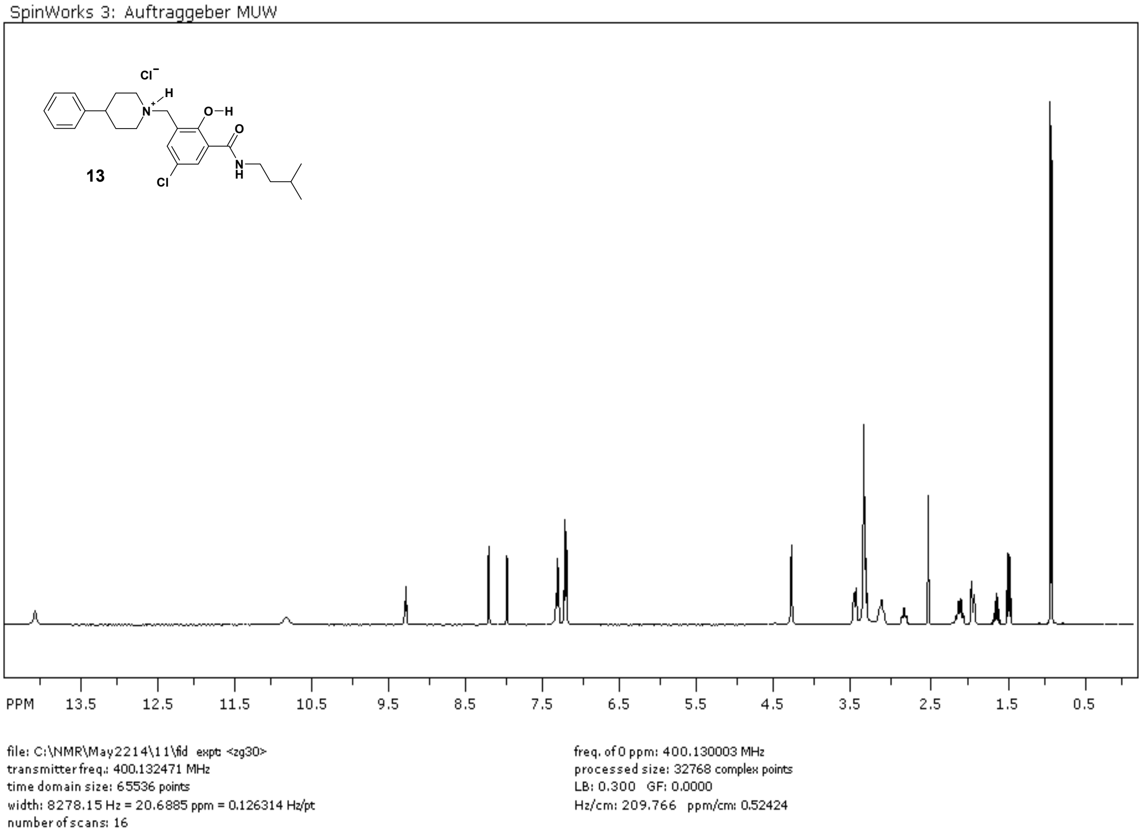

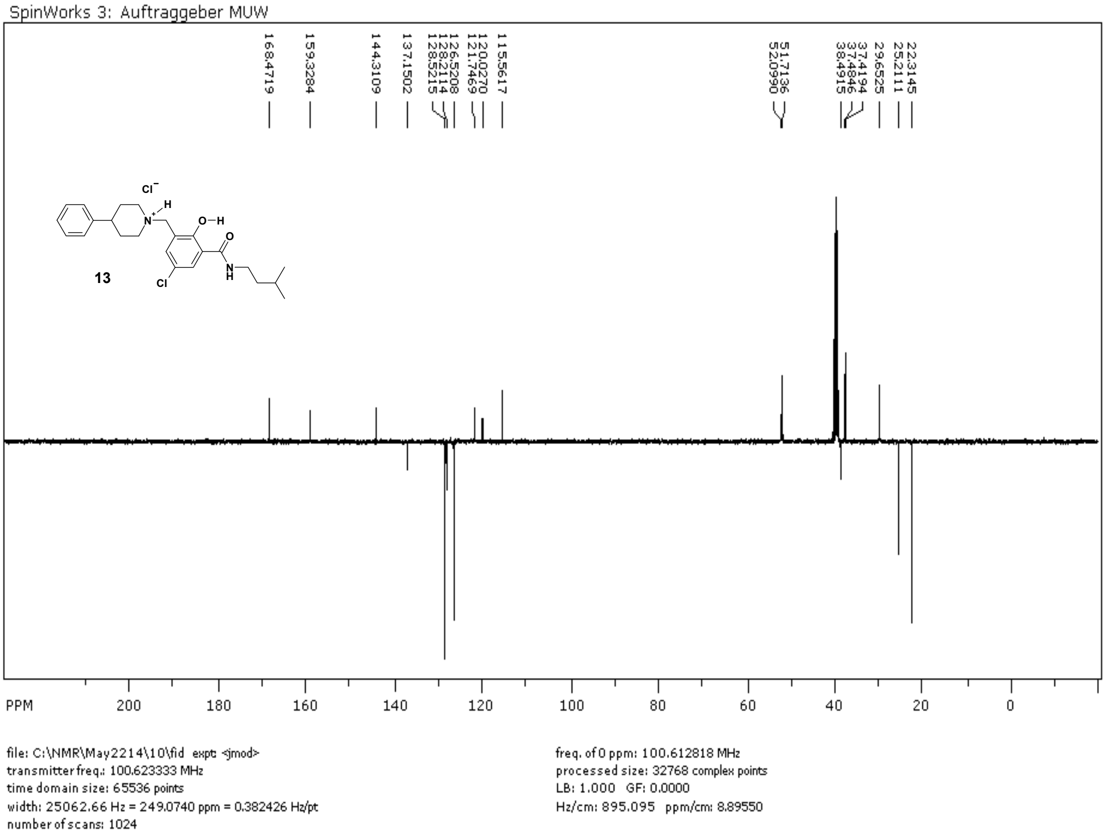

(14) Diethyl-[2-hydroxy-3-(3-methyl-butylcarbamoyl)-benzyl]-ammonium chloride

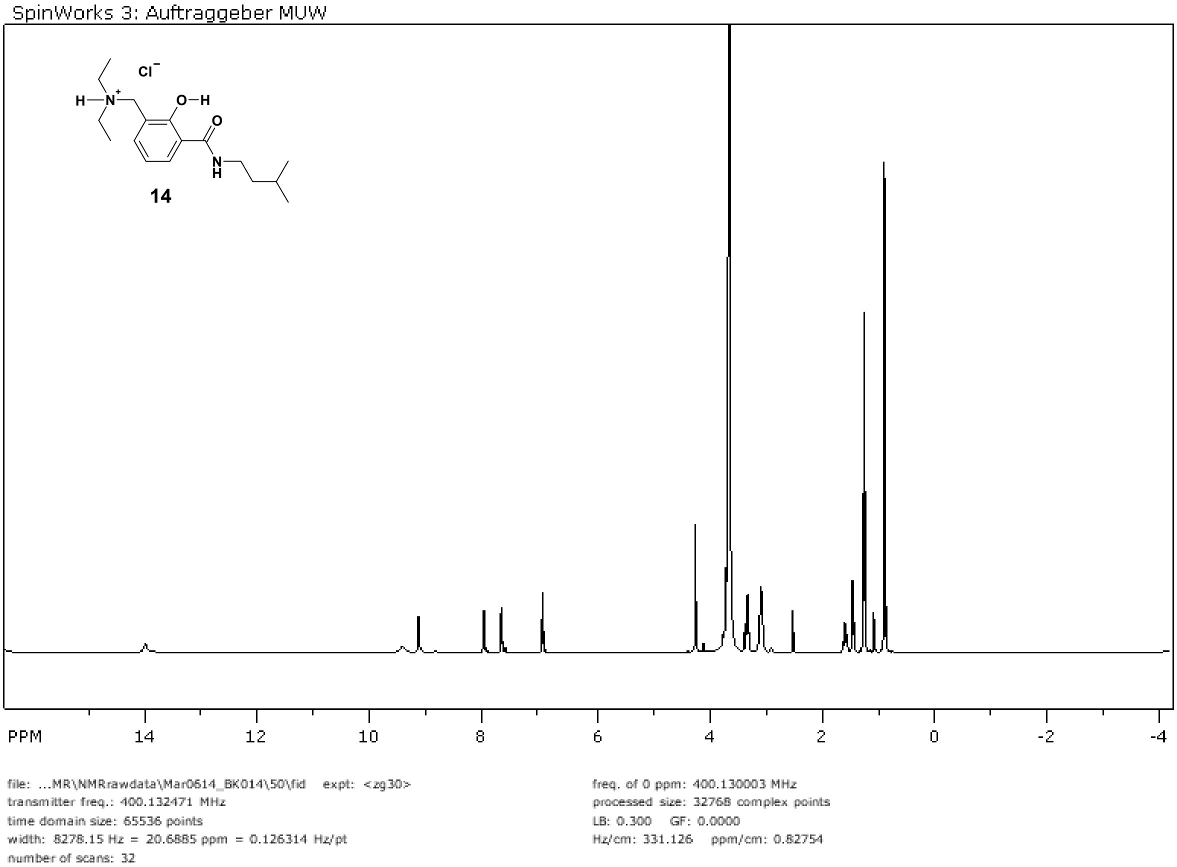

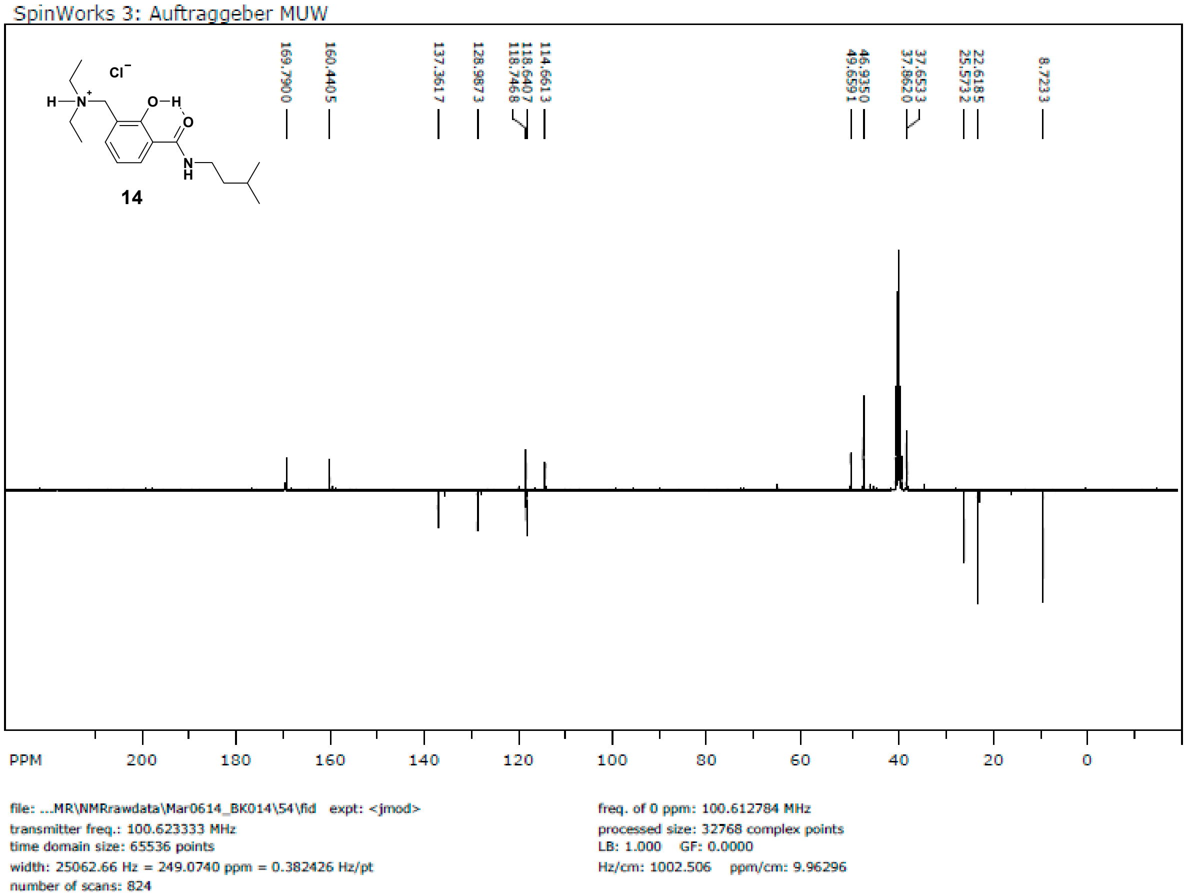

(15) [5-Chloro-2-hydroxy-3-(3-methyl-butylcarbamoyl)-benzyl]-diethyl-ammonium; chloride

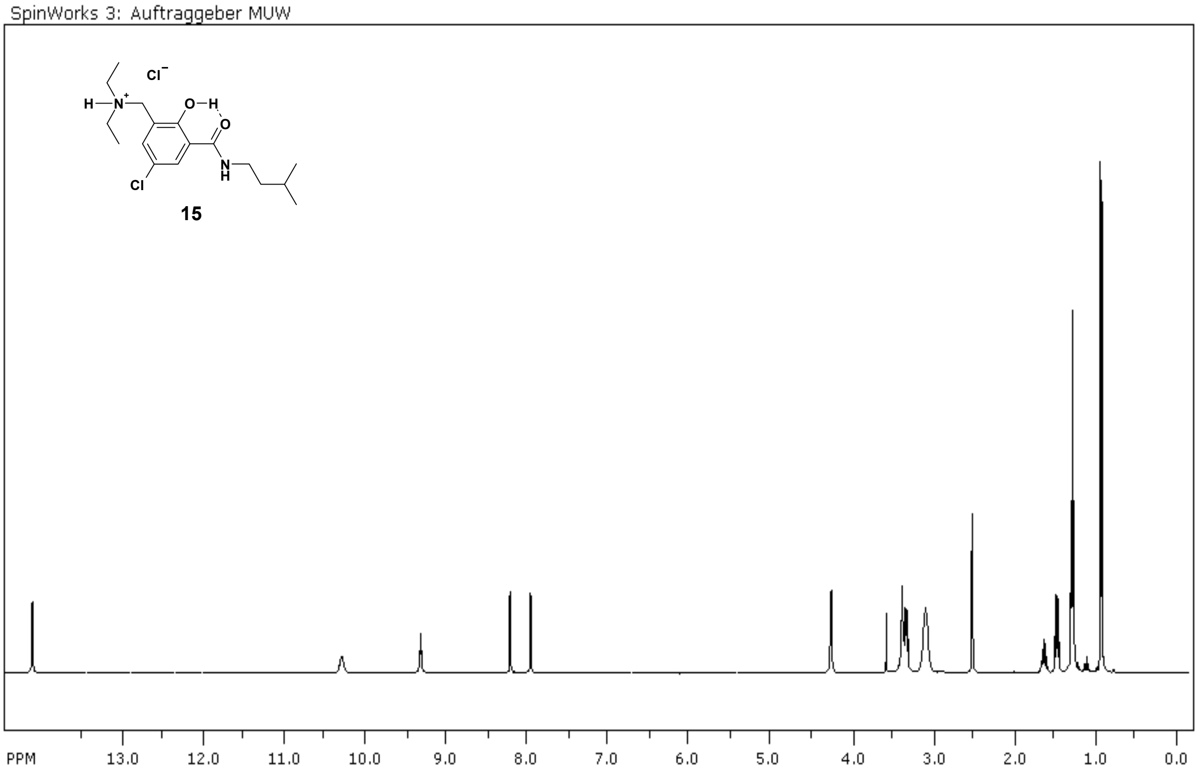

file: ...chknopf\NMR spectra\May2214\21\fid expt: <zg30>
transmitter freq.: 400.132471 MHz
time domain size: 65536 points
width: 8278.15 Hz = 20.6885 ppm = 0.126314 Hz/pt
number of scans: 16

freq. of 0 ppm: 400.130003 MHz
processed size: 32768 complex points
LB: 0.300 GF: 0.0000
Hz/cm: 209.512 ppm/cm: 0.52361

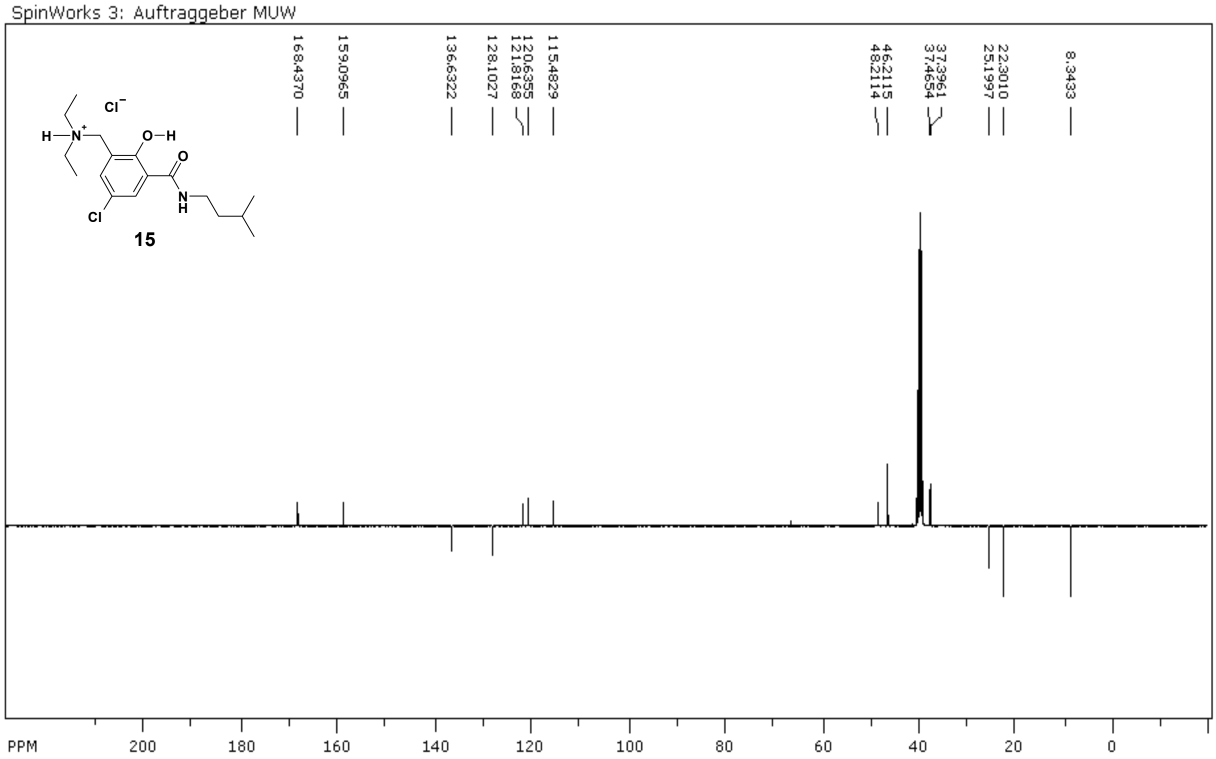

file: ...chknopf\NMR spectra\May2214\20\fid expt: <jmod>
transmitter freq.: 100.623333 MHz
time domain size: 65536 points
width: 25062.66 Hz = 249.0740 ppm = 0.382426 Hz/pt
number of scans: 2500

freq. of 0 ppm: 100.612818 MHz
processed size: 32768 complex points
LB: 1.000 GF: 0.0000
Hz/cm: 895.095 ppm/cm: 8.89550

5. Crystals Grown for Free Base 9 and Hydrochlorides 13 and 14

5-Chloro-2-hydroxy-*N*-(3-methyl-butyl)-3-(4-phenyl-piperidin-1-ylmethyl)-benzamide (**9**):

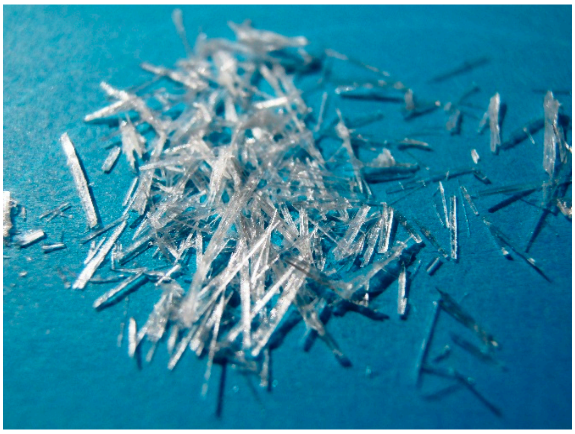

1-[5-Chloro-2-hydroxy-3-(3-methyl-butylcarbamoyl)-benzyl]-4-phenyl-piperidinium chloride (**13**):

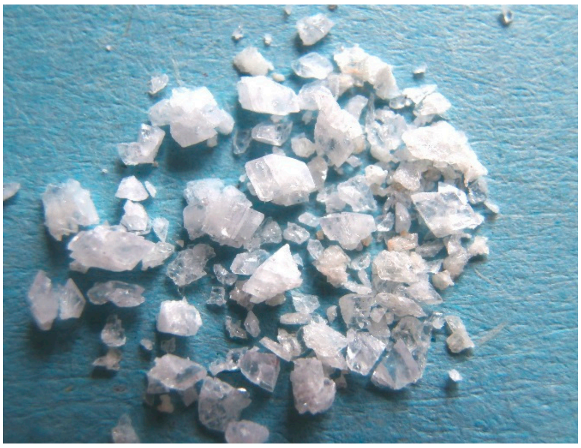

Diethyl-[2-hydroxy-3-(3-methyl-butylcarbamoyl)-benzyl]-ammonium chloride (**14**):

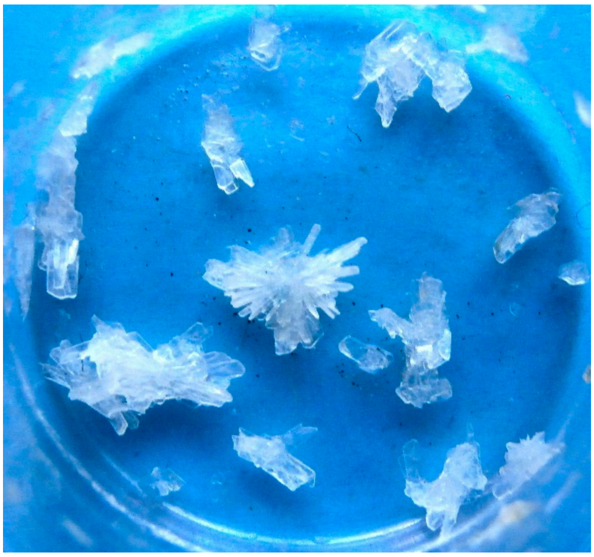

Supplement: Supplementary file 1 [file molecules-20-01686-s001.pdf]
